# Contrasting Genetic Footprints among Saharan Olive Populations: Potential Causes and Conservation Implications

**DOI:** 10.3390/plants10061207

**Published:** 2021-06-14

**Authors:** Guillaume Besnard, Océane Gorrilliot, Pauline Raimondeau, Benoit Génot, Ahmed El Bakkali, Fabien Anthelme, Djamel Baali-Cherif

**Affiliations:** 1 CNRS-UPS-ENFA, EDB, UMR 5174, Bât. 4R1, CEDEX 9, 31062 Toulouse, France; gorrilliot.oceane.pro@gmail.com (O.G.); pauline.raimondeau@univ-tlse3.fr (P.R.); benoitgenot38@gmail.com (B.G.); 2 INRA, UR APCRPG, BP 578, Meknès 50000, Morocco; ahmed.elbakkali@inra.ma; 3 AMAP, University Montpellier, IRD, CIRAD, CNRS, INRA, 34398 Montpellier, France; fabien.anthelme@ird.fr; 4 Laboratoire de Recherche sur les Zones Arides, USTHB/INA, BP44, Alger 16000, Algeria; bacherdj@yahoo.fr

**Keywords:** dry area, gene flow, isolation by distance, Laperrine’s olive, microsatellites, plastid DNA, Sahara desert, seed and pollen dispersal, wild olive genetic resources

## Abstract

The Laperrine’s olive is endemic to the Saharan Mountains. Adapted to arid environments, it may constitute a valuable genetic resource to improve water-stress tolerance in the cultivated olive. However, limited natural regeneration coupled with human pressures make it locally endangered in Central Sahara. Understanding past population dynamics is thus crucial to define management strategies. Nucleotide sequence diversity was first investigated on five nuclear genes and compared to the Mediterranean and African olives. These data confirm that the Laperrine’s olive has a strong affinity with the Mediterranean olive, but it shows lower nucleotide diversity than other continental taxa. To investigate gene flows mediated by seeds and pollen, polymorphisms from nuclear and plastid microsatellites from 383 individuals from four Saharan massifs were analyzed. A higher genetic diversity in Ahaggar (Hoggar, Algeria) suggests that this population has maintained over the long term a larger number of individuals than other massifs. High-to-moderate genetic differentiation between massifs confirms the role of desert barriers in limiting gene flow. Yet contrasting patterns of isolation by distance were observed within massifs, and also between plastid and nuclear markers, stressing the role of local factors (e.g., habitat fragmentation, historical range shift) in seed and pollen dispersal. Implications of these results in the management of the Laperrine’s olive genetic resources are discussed.

## 1. Introduction

The Sahara Desert is an area of 9,200,000 km² with occidental and oriental boundaries stretching out from the Atlantic coast to the Red Sea [1]. This hostile environment acts as a major geographic barrier between the Mediterranean Basin and tropical Africa [2,3]. Several successive dry and wet periods have, however, induced the decline and expansion of suitable habitats for living organisms, including humans [3,4]. The last important wet period ended about 6000 years ago [5,6], after which current dry conditions were established. The summits of the Saharan massifs (reaching 3415 m in Tibesti, Chad) are characterized by a less arid climate with more regular precipitation and cooler temperatures than in the surrounding plains, and consequently they act as isolated refugia for numerous species [3,7,8]. Several long-living tree species have persisted in this environment, including a few iconic endemics such as the Tassili’s cypress (*Cupressus dupreziana* A. Camus), the Nivelle’s myrtle (*Myrtus nivellei* Batt. & Trab.) and the Laperrine’s olive (*Olea europaea* L. subsp. *laperrinei.* (Batt. & Trab.) Cif.) [9,10,11,12]. Today, anthropogenic pressures locally endanger these species, more particularly the Tassili’s cypress, which is classified in the red list of IUCN [9,12]. In addition, the long generation time of tree species may limit their ability to evolve, making them vulnerable to abrupt environmental changes.

The Laperrine’s olive (*O. e. laperrinei*) is an olive subspecies endemic to the Saharan massifs [13]. Genetic studies show that this taxon is highly differentiated from its Mediterranean, Macaronesian, African and Asian relatives [14]. Phylogeographic studies suggest that it has an ancient admixed origin, probably resulting from crossing between populations from Subtropical Africa and the Mediterranean Basin [15,16]. Currently, the taxon is present in three mountain ranges: Aïr (northern Niger), Hoggar (southern Algeria), and Jebel Marra (west of Darfur, northwestern Sudan) [17,18,19]. In the Hoggar and the Aïr mountains, annual precipitation is often less than 50–100 mm, but higher precipitation amounts make Jebel Marra more favorable to their persistence and natural regeneration [3,7,19,20]. Plastid markers show that the Central Saharan and the Sudanese populations are fully differentiated (lineages E1-*l*1 (formerly L1) and E1-*l*2 (formerly L2), respectively), indicating that these populations have not been exchanging seeds for a very long time, well before the last wet phase on the Sahara [11].

The Laperrine’s olive survival is uncertain in some places, particularly in the isolated Bagzane and Egalah mountains, Aïr, Niger [20]. This may be due to several factors, such as desert expansion, increased anthropogenic pressures, very slow “turnover” of populations (mean generation time probably exceeding several centuries) and overall harsh climatic conditions [21]. There are very few opportunities for sexual reproduction, and the persistence of the taxon is mainly ensured thanks to an important clonal growth [20,22]. The isolation of populations combined with these different factors make this subspecies vulnerable to environmental fluctuations, especially in the smallest populations. In order to establish conservation programs, it is necessary to understand more precisely the past dynamics of populations. A first genetic study of Laperrine’s olive populations from Central Sahara (Hoggar, Aïr) allowed the comparison of diversity levels between different massifs and led to the identification of differentiated genetic pools [23]. Based on nuclear markers, a higher genetic diversity in Ahaggar (formerly termed “Hoggar” [23], which actually gathers Ahaggar, Tassili n’Ajjer, Tassili n’Immidir, and Tefedest) compared to the other studied massifs was revealed. The contribution of desert barriers in limiting gene dispersal between massifs was also demonstrated, but the relative importance of gene flow through seeds and pollen was not investigated due to a lack of variation in the plastid markers used in the previous study [23]. This question can now be addressed using highly polymorphic plastid markers developed from the whole plastome [24]. A few studies have been published regarding the estimation of seeds and/or pollen movements in wild olive populations by using different approaches: a first work was conducted on the Canarian olive (*O. europaea* subsp. *guanchica* P. Vargas et al.) [25], with indirect measures based on nuclear and plastid DNA data [26], whereas other studies have identified pollen donors with paternity analyses on seeds collected in wild populations [27,28,29]. These later studies demonstrated that effective pollen flow can exceed several kilometers, and may have favored gene exchanges between patches of individuals in a highly fragmented landscape.

To cope with a severe water deficit in its natural environment, the Laperrine’s olive has developed key adaptive traits limiting water loss by transpiration, such as lanceolate leaves (long and narrow) and dense abaxial peltate scales protecting stomata [18,30]. This taxon thus represents a potential genetic resource for the improvement of the cultivated olive [11], but its main gene pools still need to be prospected and evaluated in ex situ gene banks. A collection of 37 mature Laperrine’s olives from Ahaggar is currently available, and this material was recently used to resolve a controversy on the genetic determinism of the self-incompatibility system in the *O. europaea* complex [31]. This work also demonstrated that controlled hybridization with cultivated olives is feasible despite a strong flowering time shift. Interestingly, a population genetic study revealed that recent admixture between Saharan and Mediterranean olives has already occurred, notably in Tassili n’Ajjer [32]. The contribution of the Laperrine’s olive to the cultivated gene pool remains, however, very limited [32]. In addition, advanced generation of back-crosses in the Mediterranean olive was shown to produce a relatively high frequency of triploids, probably resulting from unreduced gametes in the cultivar “Dokkar” [33], which may result from genomic incompatibilities in olive hybrids.

In this study, we used an enlarged sampling of both plant material and genetic markers to refine previous genetic analyses of the Laperrine’s olive populations [23]. The current work aims to: (i) assess the diversity within a few nuclear genes in the Laperrine’s olive compared to Mediterranean and African olives; (ii) describe and compare the genetic structure among four massifs of Central Sahara (i.e., Ahaggar, Tassili n’Ajjer, Tamgak and Bagzane-Egalah), using both plastid and nuclear DNA microsatellites; (iii) evaluate the isolation by distance on different spatial scales; and (iv) estimate the contributions of pollen and seed in the global gene flow at different geographic scales. Finally, we briefly discuss the implications of our results to define adequate conservation measures and sampling strategies in order to constitute an ex situ bank of this taxon.

## 2. Material and Methods

### 2.1. Plant Material

Here, we focused on the Central Saharan populations (plastid lineage E1.*l*1 [11,34]). Four main mountain massifs were investigated: two of them in Hoggar, Algeria (Ahaggar (16 distinct sites) and Tassili n’Ajjer (one site, plus one isolated individual)) and two others in Aïr, Niger (Tamgak (three sites) and Bagzane (two sites, plus one isolated individual in Egalah)) (Table S1). In an earlier study [23], 237 genets were sampled. Additional samplings were realized in Ahaggar in 2007 [27,35], and 383 individuals (genets) are now available (after excluding clones [20,22,23]). Four non-diploid genotypes were included in this sampling (Table S1) and excluded from population genetics analyses based on nuclear microsatellites (see [35] for a discussion on putative origins of triploids). A total of 209 genets were collected in the relatively accessible Tamanrasset District (Table S1), where an especially dense sampling of Adrar Heggueghene (elevation ca. 1400–1700 m) allowed accurate characterization of the Laperrine’s olive diversity at a local scale (ca. 250 km^2^; see below). In addition, one sample from Jebel Mara (Sudan) was investigated from a seed collected in an herbarium sample (Jackson & Ramsey 2 [K]). DNA was extracted from the embryo as previously reported [36]. This individual belongs to a population characterized by plastid lineage E1*.l*2 [11,34] and was used here for the analysis of nuclear gene diversity.

### 2.2. Genetic Markers

Nuclear gene sequences are useful to detect recent admixture and also to compare patterns of nucleotide diversity among related species [36,37]. Partial gene sequences (that include one or two introns) were thus first generated on a few Laperrine’s individuals for five loci as described by [36]: *CUL4* (cullin gene), *FAD6* (plastidial delta-12 oleate desaturase gene), *MST2* (monosaccharide transporter-2 gene), *OCO* (constans-like gene), and *OEW* (lupeol synthase gene). For each locus, sequences of different alleles were obtained following the strategies previously described [36]. Nine individuals (including three triploids [35]; Table S2) were characterized, yielding a total of 21 allelic sequences (or haplotypes) for each locus. These individuals were chosen to represent the main populations of the Laperrine’s olive (i.e., four individuals from Ahaggar, two from Tassili n’Ajjer, one from Bagzane, one from Tamgak, and one from Jebel Marra; Table S2).

We then genotyped all trees with microsatellites loci (or Simple Sequence Repeats, SSRs) for population genetics analyses. Twelve nuclear SSR loci were used to characterize 382 individuals (Table S3). Nine of these loci were already used by Besnard et al. [23] (i.e., DCA03, DCA08, DCA09, DCA14, DCA15, PA(ATT)2, GAPU45, and EMO03). We analyzed three additional loci (DCA05, DCA18, and GAPU71A [38,39]) using the protocols described in [23]. A physical linkage is known for two pairs of markers [40]: GAPU45/DCA05 with a genetic distance of 5–6 cM and DCA14/DCA15 with a genetic distance of 70–80 cM. In addition, for seed dispersal investigation, we screened variation in the plastid genome that is strictly maternally inherited in the olive [41]. In the former study [23], three polymorphic plastid microsatellites (cpSSR) were used and permitted to distinguish only five haplotypes in the Central Saharan populations. Herein, 51 plastid loci (including 41 cpSSRs [24]) were used to characterize 366 genets from the Central Saharan mountains (Aïr and Hoggar; Table S3).

### 2.3. Data Analyses

#### 2.3.1. Analyses of Nuclear Gene Sequences

For each locus, the 21 allelic sequences (or haplotypes) of subsp. *laperrinei* were combined to the 60 allelic sequences previously isolated from 17 Mediterranean olives and 13 African olives in the native range [36]. All sequences were aligned with Muscle implemented in Mega v7.0 [42]. The nucleotide diversity *π* [43] was measured with Mega as the average number of substitutions per site between two haplotype sequences. This parameter was estimated separately on subspp. *europaea*, *cuspidata*, and *laperrinei*, and then on the whole sampling. The standard error of these values was estimated by bootstrapping on haplotypes (100 replicates).

The phylogenetic relationships among haplotypes were visualized by reconstructing a reduced-median network implemented in the application Network v4.112 [44]. All substitutions and indels were coded as 1/0 and considered as equivalent characters. Multi-state variations were observed at two poly-T stretches in gene *OEW*, but these two regions were excluded from our analyses because of an expected high homoplasy on these motifs (for more details see [36]).

#### 2.3.2. Analyses of Plastid DNA Haplotypes

For each individual, a plastid DNA haplotype (or chlorotype) was defined by the combination of alleles from the polymorphic loci. For the allele coding, we followed the procedure previously described [24]. The geographic distribution of chlorotypes was represented on a map. A haplotype network was also reconstructed with the reduced-median method implemented in Network [44].

#### 2.3.3. Descriptive Analyses of Population Genetic Data

First, we tested for linkage disequilibrium between all pairs of nuclear SSR loci using Fstat v2.9.3 [45]. Then, three estimators were calculated with Fstat to depict genetic diversity patterns among populations: (i) First, the total heterozygosity *H*_T_ [46] was calculated for each locus and each population: *H*_T_ = 1 − ∑ *p_i_²*, where *p_i_* is the probability to meet the allele *i* at a given locus for the whole population. For a single nuclear SSR locus, this estimator represents the average proportion of individuals that carry two different alleles. *H*_T_ is more rightly called genetic diversity in the case of haploid plastid markers. (ii) Second, the allelic richness *R*_S_ [47] was measured and allowed the comparison of the number of alleles among samples of different sizes according to the rarefaction principle [48]. (iii) And third, the effective number of haplotypes or alleles *N_e_* [49] was estimated for the plastid and nuclear markers according to the following equation: *N_e_* = 1 / ∑ *p_i_²*. A different number of alleles can lead to the same heterozygosity according to the allele frequencies. *N_e_* represents the number of alleles of equal frequency required to lead to the same heterozygosity in a population. This estimator enables the comparison between populations or subpopulations based on their most frequent haplotypes.

To test the significance of the difference in both total heterozygosity and allelic richness between populations or subpopulations, a paired Wilcoxon test was carried out with a Bonferroni correction. We particularly tested whether the allelic richness was higher in Ahaggar than in other massifs (unilateral test) as previously reported [23].

#### 2.3.4. Identification of Genetic Clusters

Populations and subpopulations used in this study are arbitrary delimited based on geographical criteria. To check the relevance of this separation, it is possible, using nuclear markers, to define different genetic clusters within the sampling without a priori on the origin of the trees. The number of genetically homogeneous clusters (*K*) in the whole dataset and separately for the Hoggar and the Aïr populations (see below) was determined using two model-based clustering methods. We first used the snapclust clustering approach of the adegenet RStudio package [50,51] and then used the clustering method implemented in Structure v2.3.4 [52]. For snapclust, we used the *K*-means method (for a number of clusters *K* ranging from one to ten at the local scale, and selected the most appropriate *K* value using the BIC (Bayesian information criterion) statistics [53] over 20 iterations per *K* value. The Structure analysis was run under the admixture model for 1,000,000 generations after a burn-in period of 200,000, assuming a correlation among allele frequencies. Analyses were run for *K* between one and ten clusters with ten iterations for each *K* value. The most likely number of clusters was determined using the ad hoc measure Δ*K* [54] with the R program [55], whereas the similarity index between ten replicates for the same *K* clusters (*H’*) was calculated with Clumpp v1.1.2 [56]. For each retained *K* value, each accession was assigned to each cluster with a posterior membership coefficient (*p*). This analysis was done on the whole sampling (377 diploid genotypes from Central Sahara) and the 226 genotypes without missing data.

#### 2.3.5. Gene Flow Patterns at Different Geographical Scales

To assess the potential effect of geographic distance on gene flow [57], we analyzed patterns of isolation by distance (IBD) on nuclear SSR data. IBD patterns were first investigated on the whole sampling and then within each massif. The pairwise kinship coefficients of Loiselle [58] and Ritland [59] were estimated with SPAGeDi v1.5 [60]. The *p*-values for 2-sized tests of regression analysis on geographic distances for each massif were calculated with 10,000 permutations. As sampling scales ranged from 6 km in Tamgak to 150 km in Ahaggar, we finally compared IBD patterns within each massif on a scale of 6 km (i.e., the maximal distance between two samples in Tamgak).

In addition, differentiation measures based on *F*_ST_ [61] were also estimated with Fstat between each pair of populations (massifs) or subpopulations (patches of trees in a given massif) on the nuclear and plastid data. These values reflect the intensity of isolation (i.e., restriction of gene flows) between populations or subpopulations. The IBD was tested at two different geographic scales, i.e., across the Central Saharan massifs (from Aïr to Hoggar) and at the massif scale (considering 15 subpopulations from Ahaggar on an area covering ca. 3000 km^2^). Rousset [62] developed a method that associates differentiation coefficients between pairs of populations with the geographic distance that separates them. The association of *F*_ST_ / (1 − *F*_ST_) with the geographic distance (or the log of distance) provides an easier interpretation of the differentiation between subpopulations. In order to test the significance of the IBD pattern for the studied species, a Mantel test was computed with Genetix [63]. In this test, samples are permuted between different locations to test the randomness of the relation between differentiation and geographic distance. The number of permutations was set to 1000.

Finally, we investigated the relative contribution of pollen and seeds in the global gene flow at three different geographic scales, i.e., across the Central Saharan massifs (ca. 200,000 km^2^), at the massif scale (Ahaggar; ca. 3000 km^2^) and at the local scale (considering five subpopulations from Adrar Heggueghene; ca. 250 km^2^, near Tamanrasset). Gene flow in plants can be estimated by indirect methods [64], but these methods do not integrate any distinction between both components of scattering, i.e., pollen and seeds. For each plant species, the dispersion by pollen or by seeds does not have the same characteristics in terms of implicated vectors (wind, insects, mammals and/or birds) and of propagation distance [65]. Ennos [26] developed a method to estimate the relative contribution of pollen and seeds in the global gene flow (*R*):

$$R=\frac{m_{P}}{m_{S}}=\frac{\left[\left(\frac{1}{F_{STn}}-1\right)\left(1+F_{IS}\right)-2\;\left(\frac{1}{F_{STc}}-1\right)\right]}{\left(\frac{1}{F_{STc}}-1\right)}$$

This estimation is based on a model of island migration, where *m_p_* represents the pollen flow and *m_s_* the seed flow, *F_STn_* the estimated differentiation based on nuclear loci, and *F_STc_* the estimated differentiation based on plastid loci. Note that, to be realistic, this approach needs several assumptions: (i) a very low mutation rate compared to migration rate, (ii) constant and equal sizes of populations, and (iii) a constant and equal migration rate between populations. The last two points represent what is called a drift/migration equilibrium. This equilibrium turns out to be rapidly reached in nature [64]. However, when the species have a long generation time and/or perturbations are quite recent, it might be necessary to check these assumptions. In order to compare results on sister subspecies, this method was also applied to data obtained on populations of wild Mediterranean olives (oleasters) [32]. Genetic data were available for 390 individuals from 45 locations distributed across the whole Mediterranean Basin. The analysis was also performed at two geographic scales: the whole Mediterranean Basin, and in three smaller regions, namely the eastern, central and western parts as previously defined [32].

## 3. Results

### 3.1. Nuclear Gene Diversity in the Laperrine’s Olive

**Figure 1:**
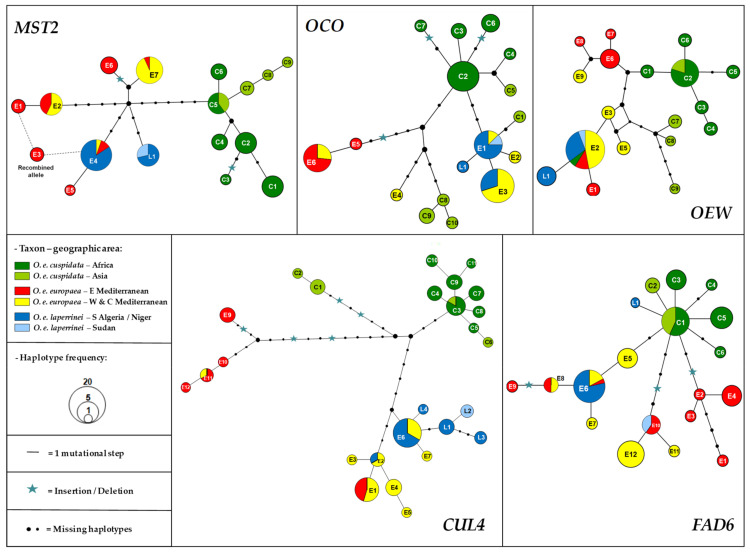
Reduced-median haplotype networks reconstructed for five nuclear loci with Network [44]. Each haplotype is numbered and represented by a pie chart. The size is proportional to the number of occurrences within our sampling. Haplotype data of Mediterranean and African wild olives are from [36]. *MST2*-E3 was identified as a recombinant allele [36]. The origin of haplotypes is indicated by a specific color.

Among the nine investigated Laperrine’s olive trees, two to six alleles were revealed at each nuclear locus (Table S2): two on *MST2* and *OEW*, three on *FAD6* and *OCO*, and six on *CUL4*. Several haplotypes were already identified in the Mediterranean olive and were named with codes used by [36]. A few new haplotypes were detected in subspecies *laperrinei* (Table S2). Those alleles (8) were then coded with L followed by a number and deposited in GenBank (EMBL numbers: LN879505 to LN879512, and OA985244 to OA985245). We thus detected one new haplotype on four loci, while four new haplotypes were detected on *CUL4*. Most nuclear gene haplotypes detected in the Laperrine’s olive are identical or closely related to haplotypes detected in the Mediterranean olive (Figure 1). Only one allele of *FAD6* (*FAD6*-L1 detected in Tassili n’Ajjer) appeared to be related to African olive haplotypes. Six of the most frequent alleles (>5 occurrences) detected in the Laperrine’s olive were shared with the Mediterranean olive (i.e., *MST2*-E4, *OLO*-E1, *OCO*-E3, *OEW*-E2, *CUL4*-E6, *FAD6*-E6; Figure 1). The individual from Jebel Marra shared alleles with the Central Saharan individuals on *MST2*, *OCO* and *OEW*, while harboring a haplotype closely related to Central Saharan haplotypes on *CUL4*. In contrast, it showed a distinct haplotype on *FAD6* (*FAD6*-E10), which was also shared with Mediterranean oleasters (Figure 1). Overall, relatively low nucleotide diversity (Table 1) was observed in the Laperrine’s olive (*π* = 0.179 ± 0.090) compared to subspecies *europaea* (*π* = 0.798 ± 0.212) and *cuspidata* (*π* = 0.459 ± 0.126 [36]).

### 3.2. Nuclear Microsatellite Polymorphism among the Four Massifs

The 12 nuclear SSRs were variable on the four studied massifs, but the number of alleles per locus strongly differed (Table S3). On the whole analyzed population, only three alleles were detected at PA(ATT)2, while 32 were found at DCA01. Otherwise, the *H*_O_ and *H*_T_ values were not significantly different regardless of the considered locus or massif (Table S4). This result indicated that there were probably no or few null alleles on the 12 loci. Note that no significant linkage disequilibrium was detected between any pair of loci, even for those that were known to be physically linked.

The mean allelic richness per locus (*R_S_* estimated for 45 samples; Table S4) ranged from 7.03 (Bagzane) to 9.13 (Ahaggar). The *R*_S_ was significantly higher in Ahaggar than in Tamgak (*Q* = 42, *p* = 0.036) and Bagzane (*Q* = 60, *p* = 0.027). The other comparisons between massifs were not significant. On the other hand, there was no significant difference between massifs in the total heterozygosity (*H*_T_), with average values ranging from 0.62 (Bagzane) to 0.67 (Tassili n’Ajjer). Finally, the highest effective number of alleles was observed in Ahaggar (*N_e_* = 4.57) and the lowest was observed in Bagzane (*N_e_* = 3.50). In Ahaggar, *H*_T_ and *R_S_* were higher in the Adrar Heggueghene subpopulations (mean *R_S_* = 11.43/72 genotypes; *H*_T_ = 0.70) than in northernmost subpopulations sampled at an elevation above 1700 m (Taessa-Tizoûadj-Akerakar; *R_S_* = 9.84; *H*_T_ = 0.67), although these values were not significantly different given the limited number of loci (i.e., estimates based on eight loci with no missing data: DCA01, DCA03, DCA05, DCA08, DCA09, DCA15, DCA18, and GAPU71A). A trend of *R_S_* reduction with increasing elevation was also revealed in all trees sampled in the Ahaggar massif (Figure S1). 

### 3.3. Plastid DNA Polymorphism and Geographic Distribution of Chlorotypes

**Figure 2:**
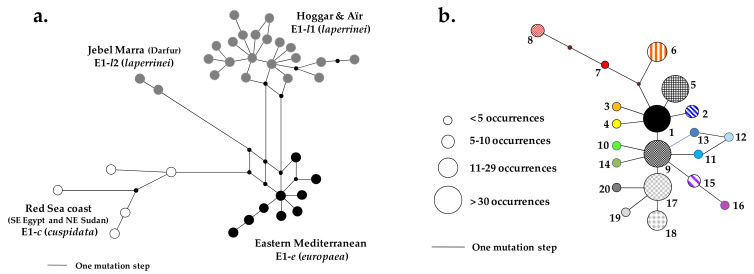
Reduced-median networks [44] of chlorotypes belonging to (**a**) lineage E1 and (**b**) sublineage E1-*l*1. Each chlorotype is represented by a circle. (**a**) Four main E1 sublineages are identified: two in Central Sahara (grey), one on the Red Sea Coast (white), and one in the eastern Mediterranean Basin (black) (modified from [11]). All Laperrine’s olive individuals from Central Sahara belong to sublineage E1-*l*1, whereas those of Jebel Marra (Darfur) belong to E1-*l*2; (**b**) On the E1-*l*1 network, the 20 chlorotypes are distinguished with a specific color and pattern (for their geographic distribution, see Figure 3). For each chlorotype, circle size is proportional to the number of occurrences within our sampling.

On the 51 tested plastid DNA markers, 15 revealed polymorphisms, enabling 20 chlorotypes to be defined (Table S5). The haplotype network (Figure 2) showed that the four most frequent chlorotypes (E1-*l*1.1, E1-*l*1.5, E1-*l*1.9 and E1-*l*1.17; frequency superior to 8%) are connected by a single mutation step and three of them were located at the center of the network. In contrast, 10 of the 15 chlorotypes with a frequency equal or inferior to 2% were at tip position, and 7 of them may derive from the most frequent chlorotypes. The relationships between E1-*l*1.11, E1-*l*1.12 and E1-*l*1.13 were not resolved and may be due to a homoplasious mutational change (either on loci 9 or 51; Table S5).

**Figure 3:**
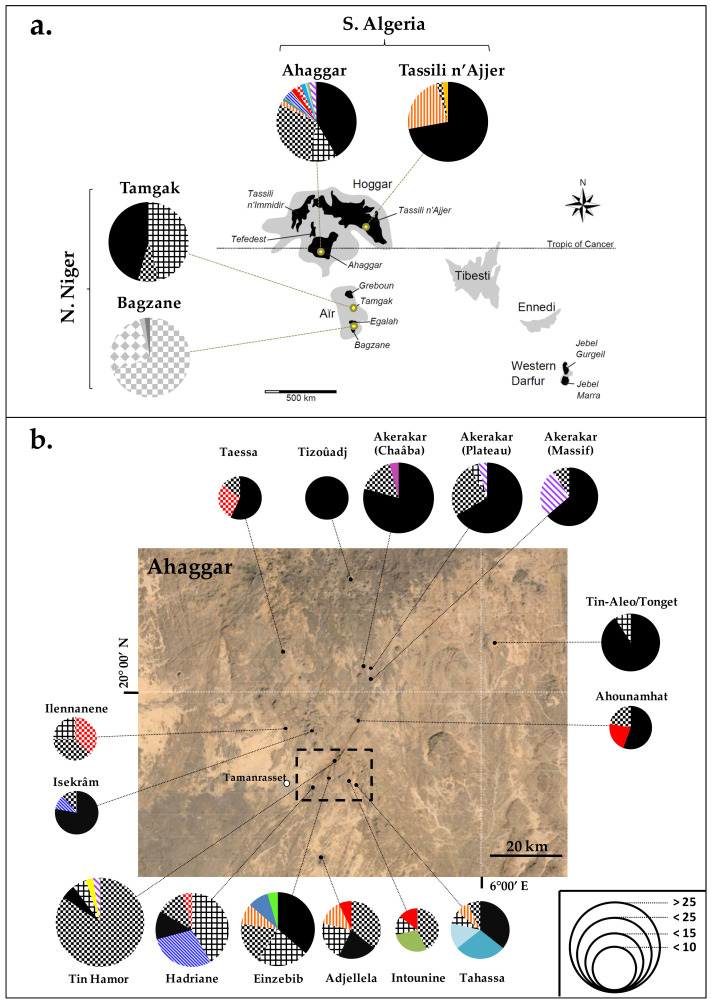
Distribution of chlorotypes in the Laperrine’s olive of Central Sahara: (**a**) in each massif sampled in Hoggar (Algeria) and Aïr (Niger), and (**b**) at each locality sampled in Ahaggar. In (**a**), the main Saharan mountains are represented in grey with the Laperrine’s olive range in black [20]. In (**b**), the area defined by the dashed lines corresponds to the most sampled area and is referred to as “Adrar Heggheguene”. The size of pie charts is proportional to subpopulation size with the scale defined by the number of individuals analyzed.

Some chlorotypes were spread over several massifs (in particular Ahaggar, Tassili n’Ajjer and Tamgak), while others were restricted to a site or a limited region (Figure 3). In Bagzane and Egalah, we found four related chlorotypes (E1-*l*1.17 to E1-*l*1.20) that were specific to this region. Individuals from Tamgak, which was geographically close to Bagzane-Egalah (70 km), harbored three frequent chlorotypes (E1-*l*1.1, E1-*l*1.5 and E1-*l*1.9) that were all shared with the Ahaggar population. This latter contained the highest plastid DNA diversity with 15 chlorotypes, among which 11 were unique to this location. Lastly, four chlorotypes (E1-*l*1.1, E1-*l*1.3, E1-*l*1.6 and E1-*l*1.9) were detected in the Tassili n’Ajjer population, but only one rare chlorotype (E1-*l*1.3; 0.2%) was unique to this massif. The allelic richness (estimated for 45 samples; Table S4) ranged from 10.97 (Ahaggar) to 3 (Tamgak). The highest values of both genetic diversity and the effective number of chlorotypes were observed in Ahaggar (*H*_T_ = 0.72, *N_e_* = 3.64), while the lowest values were found in Tassili n’Ajjer (*H*_T_ = 0.44, *N_e_* = 1.20). In Ahaggar, a particularly high plastid DNA diversity was observed in subpopulations of Adrar Heggueghene (*R_S_* = 13.53 (computed for 72 genotypes), *H*_T_ = 0.77), which contrast with subpopulations sampled at higher elevation (i.e., Taessa-Tizoûadj-Akerakar; *R_S_* = 6.00, *H*_T_ = 0.45). Such a trend of chlorotype diversity reduction with increasing elevation was also found in all the trees sampled in the Ahaggar massif (both for *R_S_* and *H*_T_; Figure S1).

### 3.4. Pattern of Genetic Differentiation and Isolation by Distance

#### 3.4.1. Genetic Differentiation between Massifs

**Figure 4:**
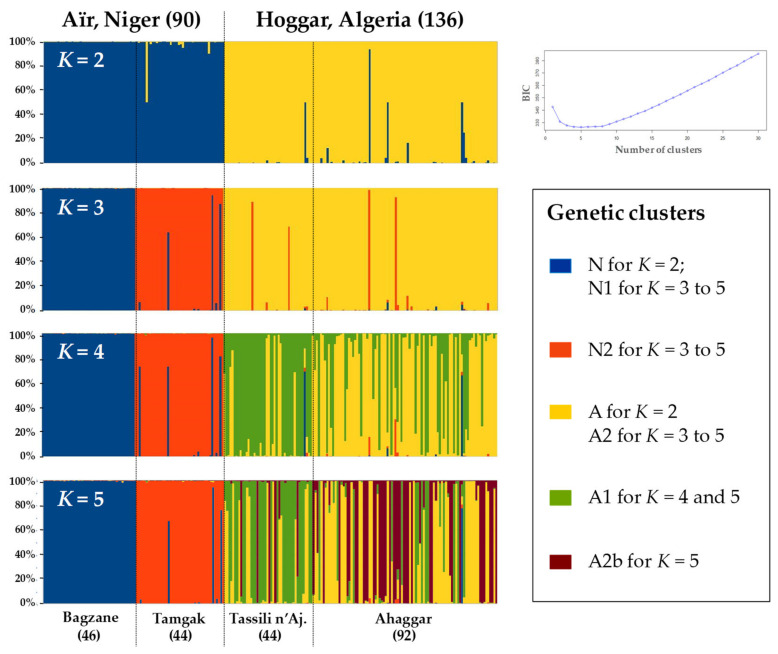
Inference of population structure using Bayesian simulations with snapclust [50] based on 226 SSR genotypes with no missing data. On the right, Bayesian Information Criterion (BIC [53]) values over 20 iterations per *K* indicate that the best *K* value is 5. On the left, barplots of the snapclust analyses based on *K* = 2 to 5 are shown. The percentage (*p*) of assignment of each individual to gene pools averaged over 20 iterations is shown. Each individual is represented by a vertical bar. The four main massifs are indicated as predefined regions. At *K* = 2, most individuals are correctly assigned to Aïr (N) or Hoggar (A) clusters. At *K* = 3, two gene pools are distinguished in Niger (N1 for Bagzane, and N2 for most individuals sampled in Tamgak). Finally, at *K* = 4 and 5, the Hoggar cluster is split into clusters (A1 and A2; or A1, A2 and A2b) that poorly match to each massif, suggesting a low differentiation between Tassili n’Ajjer (Tassili n’Aj.) and Ahaggar.

Based on nuclear markers, the populations of the four massifs were significantly differentiated (Table 2). The highest differentiation value (*F*_ST_) was detected between Tassili n’Ajjer and Bagzane (11.9%), whereas the lowest value was observed between Ahaggar and Tassili n’Ajjer (2.5%). The differentiation values were higher on plastid markers, with *F*_ST_ ranging from 9.4% between Tamgak and Ahaggar to 55.4% between Tassili n’Ajjer and Bagzane (Table 2). Bayesian clustering analyses also supported the recognition of four or five main gene pools that approximately matched each sampled massif (Figure 4, Figures S2 and S3). However, the individuals from Ahaggar and Tassili n’Ajjer appeared particularly admixed, probably a consequence of the low differentiation between these two massifs. When analyzing the Hoggar and the Aïr populations separately, we did not observe any clear structure in Hoggar, whereas only two differentiated clusters (Bagzane vs. Tamgak) were still highly supported in Aïr (data not shown).

#### 3.4.2. Isolation by Distance

**Figure 5:**
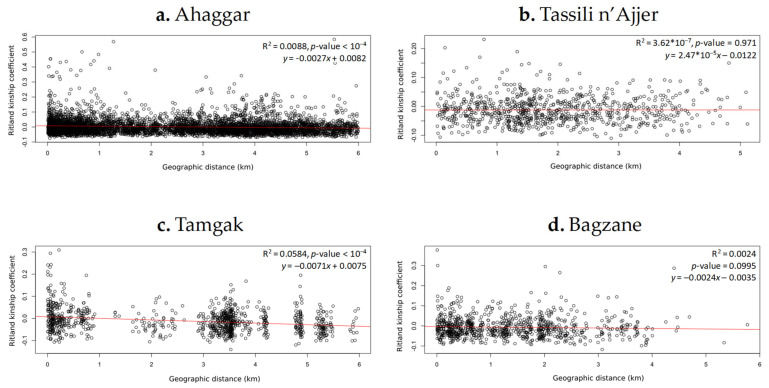
Comparison of Ritland pairwise kinship relationships [59] according to geographic distance in four Saharan massifs on a 6-km scale: (**a**) Ahaggar, (**b**) Tassili n’Ajjer, (**c**) Tamgak, and (**d**) Bagzane. The R-square and *p*-value statistics are given in the upper right. Each point represents a kinship estimate on a pair of individuals.

Patterns of IBD were first investigated on nuclear data. At the Central Sahara scale, a highly significant IBD pattern (both for Ritland and Loiselle kinship coefficients, *p*-value < 10^−4^) was observed (R^2^ ranging from 0.066 to 0.080; Figure S4). At the massif scale (Figures S5–S8), IBD was greatly reduced in the Bagzane and Hoggar massifs (and non-significant in Tassili n’Ajjer), but a relatively strong pattern (R^2^ = 0.06) was still observed in Tamgak (Figure S6). When comparing the four massifs on the same distance scale (6 km; Figure 5 and Figure S9), we confirmed a stronger IBD pattern in Tamgak than in other massifs. A very low (R^2^ < 0.01) but still significant IBD pattern was revealed in Ahaggar, probably thanks to a denser sampling in this massif.

**Figure 6:**
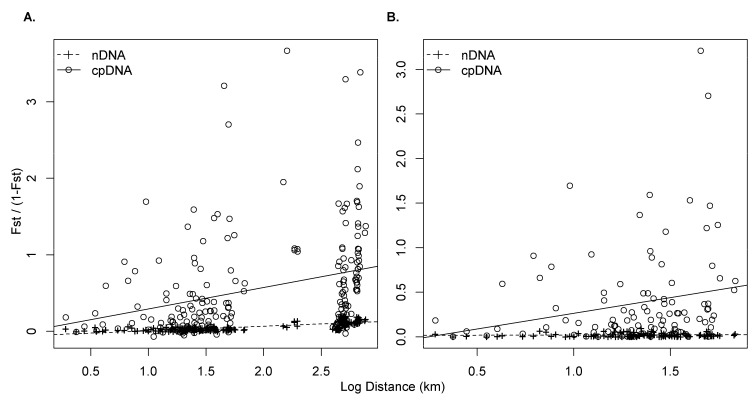
Comparative analysis of the isolation by distance according to the nuclear and plastid markers at two spatial scales: (**A**) Central Sahara (including the four massifs; 21 localities; cpDNA: *r* = 0.303, *p* = 0.01; nuclear SSRs (nDNA): *r* = 0.153, *p* = 0.01), and (**B**) Ahaggar (15 localities; cpDNA: *r* = 0.209, *p* = 0.10; nuclear SSRs (nDNA): *r* = 0.143, *p* = 0.17). *F*_ST_ / (1 − *F*_ST_) was plotted against the logarithm of geographic distance following Rousset [62].

Based on *F*_ST_ values, an IBD pattern was also revealed over the Central Saharan mountains. This relation was stronger for plastid DNA (*r* = 0.303, *p* = 0.01) than for nuclear loci (*r* = 0.153, *p* = 0.01; Figure 6a). The IBD pattern did not remain significant at the local scale in the Ahaggar massif (Figure 6b; Table S6).

#### 3.4.3. Pollen vs. Seed Flow

The estimated contributions of pollen and seed dispersal to gene flow are summarized in Table 3. The relative contribution of pollen was higher than the seed contribution by about one order of magnitude (from 6.37 to 33.28). This disparity increased when the geographic scale decreased. Thus, the highest *R* value was observed at the local scale, in Adrar Heggueghene (*R* = 33.28), and was intermediate at the massif scale (*R* = 17.08 in Ahaggar). In the Mediterranean Basin, a similar trend was revealed, since *R* reached 7.06 on the whole area, while the value tended to increase both in the western and central regions (*R* = 39.99 and 16.22, respectively) but remained similar in the eastern area (*R* = 6.36; Table 3).

## 4. Discussion

This work represents an update of the study published by [23]. We report new data on the nucleotide diversity of single-copy genes and extend the microsatellite dataset especially for plastid markers. These data enable us to better depict patterns of genetic diversity, taking advantage of two differentially inherited genome compartments, and to make some comparisons with other continental olive taxa. These investigations reveal particularly low nucleotide diversity in the Laperrine’s olive but contrasting patterns of differentiation and isolation by distance within massifs, and also between plastid and nuclear markers. Our observations may have implications for in situ conservation of this endangered taxon in the Saharan mountains, but also for its management in ex situ collections to evaluate its potential value as a genetic resource for the improvement of the cultivated olive.

### 4.1. Low Nucleotide Diversity in Nuclear Genes of the Laperrine’s Olive

Relatively low variation (*π* = 0.179) was found in five nuclear gene regions among nine trees sampled over the whole distribution area of the Laperrine’s olive (Table 1). This contrasts with other continental olive subspecies that show nucleotide diversity more than twice as high. This may be due to the strong isolation of Laperrine’s olive populations in the Sahara Desert that should favor genetic drift. In addition, although similar to trees sampled in Hoggar and Aïr, the individual of Jebel Marra shows different alleles on two loci, supporting the hypothesis that the Central Saharan and Northwest Sudanese populations have evolved independently for a long time (as also indicated by plastid DNA markers [11]). In previous works, nuclear ribosomal DNA analyses (based on both functional units and pseudogenes) as well as SSR data rendered contrasting results indicating that the Saharan populations have been recurrently admixed with Mediterranean and African olives [16,30,32]. Here, a strong affinity between all the Laperrine’s olive populations with the Mediterranean olives was supported by haplotype networks (Figure 1), in accordance with plastid DNA markers [14,15,16,30]. Only one allele of *FAD6* (*FAD6*-L1, in the individual of Jabbaren, Tassili n’Ajjer) supports the hypothesis of gene introgression from subsp. *cuspidata* into the Laperrine’s olive gene pool, but the contribution of this taxon seems minor in comparison to the Mediterranean olive.

### 4.2. Is Ahaggar a Long-Lasting Region for Laperrine’s Olive Survival?

The population genetic study was then focused on Central Saharan olives that are characterized by the plastid lineage E1.*l*1 (Figure 2a). In a previous study, nuclear SSR data suggested that populations from the Ahaggar massif harbor a higher diversity than Niger populations [23]. Here, both plastid and nuclear DNA data strongly sustain that Ahaggar is a hotspot of genetic diversity of the Laperrine’s olive in Central Sahara. In particular, plastid allelic richness was more than twice as high in Ahaggar than any other population analyzed in the present study (Table S4), even Tassili’n Ajjer where genetic admixture with western Mediterranean olives has occurred within the past 10 millennia [32]. Our results indicate that the Ahaggar population was able to maintain a high diversity, especially at lower elevation near Tamanrasset (Figure 3 and Figure S1). This is probably due to favorable climatic conditions and/or a large suitable habitat that prevailed in this massif and allowed a large population to maintain over a long period. This population may have also acted as a putative source for colonizers of the other massifs, particularly Tamgak and Tassili n’Ajjer, which both share major chlorotypes with Ahaggar (Figure 3a). However, individuals of Bagzane do not share any chlorotype with other populations, indicating no recent seed exchange as previously stated [23].

In the Ahaggar massif, a high differentiation between subpopulations separated by a few kilometers was observed based on cpDNA markers (Figure 3b; Table S5). This observation sustains that gene flow through seeds between patches was limited in the past. Interestingly, a reduction of gene diversity with elevation also suggests that the altitudinal distribution range of the Laperrine’s olive shifted during climatic changes of the Quaternary. In the Hoggar Mountains, palaeobotanical records (and in particular macrofossils) of the Last Glacial Maximum and the Holocene attest that the lower elevation limit for olive persistence was inferior (<1000 m) than currently (ca. 1400 m) [66,67]. Interestingly, most of the chlorotypes detected in Ahaggar were revealed in Adrar Heggueghene (<1700 m). Wadis located at 1400–1700 m were certainly suitable habitats for the olive during cold periods in contrast to higher elevation [3,4,66,68]. Long-distance spreading of the olive in the highest areas during interglacials should have been limited due to few dispersal vectors, especially for fruits (e.g., frugivorous birds) that can pass geographic barriers such as dry areas (which cover most parts of the Saharan mountains) and high mountain tops (higher than 2500 m in Ahaggar). It is also conceivable that humans occasionally participated in the spread of the tree, which represents a source of wood and forage for local populations [11,67]. The relatively low cpDNA diversity in trees spread through a network of temporary rivers above 1700 m is therefore the consequence of a limited number of founders via seeds, and/or long-persistence of few individuals during unfavorable periods leading to strong genetic drift on maternally inherited genomes.

### 4.3. Contrasting Patterns of Isolation by Distance among Massifs, Spatial Scales and Genomes

The IBD pattern revealed in Central Sahara (Figure 6 and Figure S4) mostly reflects the strong genetic differentiation between main populations, as also shown in Nivelle’s myrtle [8]. At the massif scale, IBD patterns based on nuclear SSRs are very reduced but still significant in three of the four populations studied (i.e., Ahaggar, Tamgak and Bagzane; Figures S5–S8). At a short scale (6 km; Figure 5), a relatively strong IBD was revealed in Tamgak, confirming a previous analysis [23]. This pattern could result from single colonization in a strongly constrained network of wadis, where our sampling consisted of three main patches of individuals that may be locally related. In the Ahaggar massif, the trend of IBD was more pronounced on plastid markers than on nuclear SSRs. This pattern is expected since, as mentioned above, seed dispersal may be very limited compared to pollen that is spread by wind. Pollen flow contribution to global gene flow, however, strongly differs according to the considered scale (*R* = 33.28 in Adrar Heggueghene vs. 6.37 in Central Sahara; Table 3). Our results indicate that the relative effects of distance on gene dispersal limitations may increase faster for pollen than seeds. A similar trend was observed in oleaster populations, although no decrease of *R* was revealed in the eastern Mediterranean region (Table 3). Interestingly, García-Verdugo et al. [25] reported an *R* value of 1.64 in olive populations of subsp. *guanchica*, suggesting that seeds have a higher relative contribution to gene flow in the Canary Archipelago than in the Saharan mountains and the Mediterranean Basin. However, as the distance scale considered seems to strongly influence *R*, a similar spatial framework should be used for a formal comparison between taxa.

At the scale of Adrar Heggueghene, a low and nonsignificant geographic structure was revealed based on nuclear SSRs (Table S6), suggesting the existence of numerous exchanges during reproduction phases. The high *R* value might reflect the huge importance of pollen gene flow at this scale, and also a limited dispersal of seeds. On a larger scale, gene flow probability should be much reduced, and this could decline with the distance faster for pollen than for seeds, possibly explaining our results. However, at the largest spatial scale, some massifs may not have exchanged individuals for a long time, whereas peripheral populations may have been impacted by strong genetic drift (e.g., Bagzane, with a current population of ca. 100 trees). Moreover, measures of differentiation on each genome could be differently biased due to variable mutation and homoplasy rates [69,70]. In addition, seed dispersal may have strongly varied over time [71] depending, in particular, on the environmental conditions and the presence of frugivorous birds. Consequently, caution should be taken when interpreting such analyses, and direct estimations of gene flow are still necessary to confirm our observations [72]. But until now, no saplings have been observed in Central Sahara, and only pollen flow estimation should be possible in the field [27].

## 5. Conclusions

Knowledge of the genetic structure of olive populations and their dispersal ability is key to identify taxonomic units or gene pools and then to define appropriate conservation actions [73]. Population genetics has already been used to investigate gene flow in wild olives, such as in anthropogenically disturbed highlands of Ethiopia and Oman [74,75,76]. These studies allowed setting up conservation actions for limiting genetic erosion due to inbreeding in the fragmented landscapes [74,76]. In the Saharan mountains, the anthropogenic pressures are particularly worrying given that natural olive regeneration has never been recorded in this area [20,22]. As stated previously for the Bagzane population [23], the genetic uniqueness of endangered Niger populations makes their protection and sampling highly relevant. In situ management of such taxa might require reinforcing populations. Restoration practices to manage relictual populations should be mostly based on seedlings coming from the same mountain range to maintain the genetic integrity of populations and their potential local adaptation [71]. An ethnobotanical survey is necessary to appreciate the putative role played by local populations (Touaregs) in the dispersal of Saharan trees in the past and present times. 

These genetic resources should also be conserved in gene banks. Individuals of each main gene pool have to be collected to get a representative sampling of the diversity of the Laperrine’s olive. Until now, the only collection of Laperrine’s olive was constituted with seedlings sampled from very few places in the Ahaggar massif (i.e., Adjellela, Tin Hamor, Hadriane, Akerakar, Tonget, and Tizoûadj). The genetic diversity of this collection was, however, shown to be significantly reduced when compared to the initial population, as the consequence of seeds collected on a limited number (12) of mother trees [27,31]. To limit such a genetic impoverishment, seeds should be collected on more mother trees from large populations, especially during years of huge flowering to favor mutipaternity [27]. Alternatively to seedlings, cuttings may be preferred to propagate genotypes subjected to natural selection. Sampling in all the Saharan massifs, and especially in the foothills of Ahaggar where a high genetic diversity has been observed, is still necessary to constitute a reference of living collections of Laperrine’s olive that could be evaluated for some agronomical traits such as drought tolerance and vigor, and be used to produce hybrids with the cultivated olive [31]. An exhaustive sampling would also include natural hybrids from Tassili n’Ajjer [32].

## Figures and Tables

**Table 1:** Estimates of nucleotide diversity (*π* in % [43]) in five gene segments (loci) among three olive taxa. *π* is given for each locus and on the total dataset. *n* is the number of sequences (haplotypes) used for each taxon. The standard error of these values was estimated with bootstrapping on haplotypes (100 replicates). Estimates for the Mediterranean (*europaea*) and African (*cuspidata*) olives are based on sequences isolated from 17 and 13 native wild olives, respectively [36].

Taxa	*n*	Locus					Mean
		*MST2*	*OCO*	*OEW*	*CUL4*	*FAD6*	
Subsp. *laperrinei*	21	0.373 ± 0.162	0.076 ± 0.056	0.099 ± 0.100	0.144 ± 0.063	0.260 ± 0.101	0.179 ± 0.090
Subsp. *europaea*	34	0.766 ± 0.261	0.798 ± 0.209	0.521 ± 0.155	1.021 ± 0.200	0.732 ± 0.243	0.798 ± 0.212
Subsp. *cuspidata*	26	0.520 ± 0.167	0.443 ± 0.105	0.817 ± 0.198	0.390 ± 0.105	0.200 ± 0.081	0.459 ± 0.126
All three taxa	81	1.393 ± 0.325	0.717 ± 0.195	0.799 ± 0.196	1.007 ± 0.187	0.603 ± 0.181	0.899 ± 0.210

**Table 2:** Pairwise genetic differentiation (*F*_ST_; in percent) between populations (massifs) based on nuclear SSR markers (nDNA) and plastid markers (cpDNA).

Massif	Ahaggar		Tassili n’Ajjer		Tamgak	
	nDNA	cpDNA	nDNA	cpDNA	nDNA	cpDNA
Tassili n’Ajjer	2.5 *	13.1 *				
Tamgak	9.1 *	9.4 *	8.8 *	23.4 *		
Bagzane	9.8 *	37.2 *	11.9*	55.4 *	8.1 *	47.7 *

Significance values for differentiation tests among populations are based on 6000 permutations; * *p* < 0.001.

**Table 3:** Parameter values used in the Ennos’s equation [26] in different geographic areas in order to estimate the pollen to seed ratio (*R*) in populations of two olive subspecies (*laperrinei* and *europaea*): *N*_pops_ represents the number of subpopulations, *F*_ST*c*_ and *F*_ST*n*_ represent the differentiation value based respectively on plastid and nuclear polymorphisms, *F*_IS_ represents the level of inbreeding. Three geographic scales were considered for the Laperrine’s olive (i.e., Central Sahara, Ahaggar, Adrar Heggueghene), whereas three main regions were delimited in the Mediterranean Basin (data from [32]).

Subspecies	Geographic Area	*N*_pops_	*F*_ST*c*_	*F*_ST*n*_	*F*_IS_	*R*
*laperrinei*	Central Sahara	21	0.376	0.061	−0.002	6.37
	Ahaggar	15	0.294	0.017	−0.019	17.08
	Adrar Heggueghene	5	0.319	0.012	−0.001	33.28
*europaea*	Whole Mediterranean Basin	45	0.499	0.098	−0.012	7.06
	Western part	15	0.545	0.061	−0.012	16.22
	Central part	13	0.719	0.057	−0.008	39.99
	Eastern part	17	0.375	0.066	−0.015	6.36

## Data Availability

All samples information and genotyping data are provided in Supplemental Data (Table S3). All nuclear gene haplotypes have been deposited in GenBank under accession nos LN879505 to LN879512, and OA985244 to OA985245.

## Supplementary Materials

The following are available online at https://www.mdpi.com/article/10.3390/plants10061207/s1. Table S1. List of populations characterized in the present study; Table S2. Haplotypes revealed at five single copy-genes in nine Laperrine’s olive individuals; Table S3. Complete dataset, including genetic profile (nuclear SSR profile + chlorotype), GPS coordinates, massif, population and subpopulation for each studied tree; Table S4. Genetic diversity and microsatellite summary statistics by sampling site; Table S5. cpSSR profile and frequency of the 20 haplotypes detected in central Sahara based on 15 polymorphic plastid loci; Table S6. Pairwise FST values between the 15 Ahaggar subpopulations based on plastid haplotypes and 12 nuclear SSRs; Figure S1. Mean allelic richness and gene diversity according to elevation within the Ahaggar massif assessed on chlorotypes and nuclear microsatellites; Figure S2. Inference of population structure on the whole dataset (377 diploid genotypes) using model-based Bayesian clustering implemented in Structure; Figure S3. Inference of population structure on 226 individuals with no missing data using model-based Bayesian clustering implemented in Structure; Figure S4. Pairwise kinship relationships according to geographic distance in Laperrine’s olives of Central Sahara; Figure S5. Pairwise kinship relationships according to geographic distance in Laperrine’s olives of the Ahaggar massif; Figure S6. Pairwise kinship relationships according to geographic distance in Laperrine’s olives of the Tassili n’Ajjer massif; Figure S7. Pairwise kinship relationships according to geographic distance in Laperrine’s olives of the Tamgak massif; Figure S8. Pairwise kinship relationships according to geographic distance in Laperrine’s olives of the Bagzane massif; Figure S9. Comparison of pairwise kinship relationships [58] according to geographic distance in four Saharan massifs on a 6-km scale.
