# Supplementary material for: Contrasting Genetic Footprints among Saharan Olive Populations: Potential Causes and Conservation Implications
Source: Plants (Basel). 2021 Jun 14;10(6):1207. doi: 10.3390/plants10061207 (PMC8231981; doi:10.3390/plants10061207)
Supplement: Supplementary file 1 [file plants-10-01207-s001.zip › Supplementary information v5.pdf]

Supplementary information

Contrasting Genetic Footprints among Saharan Olive Populations: Potential Causes and Conservation Implications

G. Besnard, et al.

Supplementary information includes:

Table S1. List of populations characterized in the present study

Table S2. Haplotypes revealed at five single-copy genes in nine Laperrine's olive individuals

Table S3. Complete dataset, including genetic profile (nuclear SSR profile + chorotype), GPS coordinates, massif, population and subpopulation for each studied tree

Table S4. Genetic diversity and microsatellite summary statistics by sampling site

Table S5. cpSSR profile and frequency of the 20 haplotypes detected in central Sahara based on 15 polymorphic plastid loci

Table S6. Pairwise F_{ST} values between the 15 Ahaggar subpopulations based on plastid haplotypes and 12 nuclear SSRs

Figure S1. Mean allelic richness and gene diversity according to elevation within the Ahaggar massif assessed on chlorotypes and nuclear microsatellites

Figure S2. Inference of population structure on the whole dataset (377 diploid genotypes) using model-based Bayesian clustering implemented in STRUCTURE

Figure S3. Inference of population structure on 226 individuals with no missing data using model-based Bayesian clustering implemented in STRUCTURE

Figure S4. Pairwise kinship relationships according to geographic distance in Laperrine's olives of Central Sahara

Figure S5. Pairwise kinship relationships according to geographic distance in Laperrine's olives of the Ahaggar massif

Figure S6. Pairwise kinship relationships according to geographic distance in Laperrine's olives of the Tassili n'Ajjer massif

Figure S7. Pairwise kinship relationships according to geographic distance in Laperrine's olives of the Tamgak massif

Figure S8. Pairwise kinship relationships according to geographic distance in Laperrine's olives of the Bagzane massif

Figure S9. Comparison of pairwise kinship relationships (Loiselle et al. 1995) according to geographic distance in four Saharan massifs on a 6-km scale

References

Table S1. List of locations characterized in the present study. The locality of sampling, the number of genotyped individuals (N) and the GPS coordinates of the location are given. See Table S3 for GPS coordinates of each individual. The number of individuals analyzed with nuclear SSRs (nSSR) and plastid SSRs (cpSSR) is given for each location.

Locality, mountain range	Country	N (nSSR)	N (ccpSSR)	Lat.	Long.
Einzebib, Ahaggar [a]	Algeria	22	22	22.801	5.624
Intounine, Ahaggar [a]	Algeria	7*	7	22.789	5.693
Tahassa, Ahaggar [a]	Algeria	14	14	22.798	5.677
Hadriane, Ahaggar [a]	Algeria	24*	24	22.783	5.590
Tin-Hamor, Ahaggar [a]	Algeria	42*	39	22.838	5.635
Akerakar « chaâba » (1), Ahaggar	Algeria	24	24	23.052	5.707
Akerakar « plateau » (2), Ahaggar	Algeria	24	24	23.043	5.728
Akerakar « massif » (3), Ahaggar	Algeria	11	11	23.021	5.715
Ahounamhat, Ahaggar	Algeria	9	9	22.932	5.699
Ilennanene, Ahaggar	Algeria	6	6	22.915	5.523
Isekrâm (Oulet), Ahaggar	Algeria	10	9	22.912	5.588
Tin-Aleo/Tonget, Ahaggar	Algeria	12	12	23.100	6.028
Adjelella, Ahaggar	Algeria	14	14	22.631	5.608
Taessa, Ahaggar	Algeria	7	7	23.085	5.520
Tizoûadj, Ahaggar	Algeria	6	6	23.244	5.679
Afilal, Ahaggar	Algeria	1	1	23.145	5.730
Inerjiounene, Ahaggar	Algeria	2	2	23.867	6.023
Assekrem, Ahaggar	Algeria	2	0	na	na
Vallée des Cyprès, Tassili n'Ajjer	Algeria	45	42	24.612	9.583
Jabbaren, Tassili n'Ajjer	Algeria	1	1	24.465	9.715
Faille du Tamgak « Ouest », Tamgak	Niger	21	21	19.500	8.617
Faille du Tamgak « Centre », Tamgak	Niger	16	17	19.500	8.650
Faille du Tamgak « Sud », Tamgak	Niger	8	8	19.167	8.650
Bagzane « Nord », Bagzane	Niger	43*	37	17.833	8.750
Bagzane « Centre », Bagzane	Niger	9	8	17.733	8.750
Egalah, Bagzane	Niger	1	1	18.175	8.699

* Including one polyploid individual that was excluded in some analyses; [a] Localities of 'Adrar Heggueghene'.

Table S2. Haplotypes (alleles) revealed at five single-copy genes in nine Laperrine's olive individuals. Alleles were coded following Besnard and El Bakkali (2004). Eight new haplotypes were detected in subspecies *laperrinei*. These sequences were then coded with L followed by a number and deposited in GenBank (EMBL numbers: LN879505 to LN879512, and OA985244 to OA985245). Three individuals were previously reported to be (auto)triploids (3n; Besnard & Baali-Cherif 2009), and three alleles were recorded at each locus. For these individuals, when two distinct alleles were present, relative intensity of peaks in sequence chromatograms allowed us to define which allele was present in double. When necessary, alleles were also separately sequenced after cloning (methods described in Besnard and El Bakkali 2014).

**Locus**	**Locus size (bp)**	**Individuals**								
		**Hadriane 6 (Ahaggar)**	**Tin-Hamor 9 (Ahaggar; 3n)**	**Hadriane 19 (Ahaggar; 3n)**	**Intounine 1 (Ahaggar; 3n)**	**VC2 (Tassili)**	**Jabbaren (Tassili)**	**Tamg. 89 (Tamgak)**	**Bagz. 81 (Bagzane)**	**JR2-emb7 (Sudan)**
MST2	516	*MST2*-E4<br>"	*MST2*-L1<br>"<br>*MST2*-E4	*MST2*-E4<br>"<br>"	*MST2*-E4<br>"<br>"	*MST2*-L1<br>*MST2*-E4	*MST2*-E4<br>"	*MST2*-E4<br>"	*MST2*-E4<br>"	*MST2*-L1<br>"
OCO	694	*OCO*-E1<br>"	*OCO*-E1<br>*OCO*-E3<br>"	*OCO*-E1<br>"<br>*OCO*-L1	*OCO*-E1<br>"<br>*OCO*-E3	*OCO*-E1<br>"	*OCO*-E1<br>"	*OCO*-E3<br>"	*OCO*-E1<br>*OCO*-E3	*OCO*-E1<br>"
OEW	524	*OEW*-L1<br>*OEW*-L2*	*OEW*-L1<br>*OEW*-L2<br>"	*OEW*-L2<br>"<br>*OEW*-L2b*	*OEW*-L1<br>"<br>*OEW*-L2	*OEW*-L1<br>"	*OEW*-L2<br>*OEW*-E2	*OEW*-L1<br>"	*OEW*-L1<br>*OEW*-E2	*OEW*-E2<br>"
CUL4	918	*CUL4*-L4<br>*CUL4*-E6	*CUL4*-L1<br>"<br>*CUL4*-E6	*CUL4*-L1<br>*CUL4*-E2<br>*CUL4*-E6	*CUL4*-E6<br>"<br>"	*CUL4*-E6<br>"	*CUL4*-L3<br>*CUL4*-E6	*CUL4*-L1<br>*CUL4*-E6	*CUL4*-E6<br>"	*CUL4*-L2<br>"
FAD6	549	*FAD6*-E6<br>"	*FAD6*-E6<br>"<br>"	*FAD6*-E6<br>"<br>"	*FAD6*-E6<br>"<br>"	*FAD6*-E6<br>"	*FAD6*-L1<br>*FAD6*-E6	*FAD6*-E6<br>"	*FAD6*-E6<br>"	*FAD6*-E10<br>"

* Note that *OEW*-L2 and -L2b are variants of *OEW*-E2, being distinguished by the addition of one or two T in a poly-T stretch (homoplasious information not taken into account in the network reconstruction; see Besnard & El Bakkali 2014).

Table S4. Genetic diversity and microsatellite summary statistics by sampling site. We give the total number of observed alleles (N_a) on the whole sample for each locus. For each sampling site, allelic richness (R_S; estimated for 45 diploid genotypes), observed heterozygosity (H_O), genetic diversity (H_T), effective number of haplotypes (N_e) estimated from nuclear SSR data (nDNA) and plastid haplotypes (cpDNA) are given for each locus and on average.

**Locus**	N_a	**Ahaggar**					**Tassili n'Ajjer**					**Tamgak**					**Bagzane**				
		N	R_s	H_O	H_T	N_e	N	R_s	H_O	H_T	N_e	N	R_s	H_O	H_T	N_e	N	R_s	H_O	H_T	N_e
**nDNA**																					
DCA05	26	25	16.5	0.88	0.88	8.3	11	10.9	0.89	0.82	5.3	12	12.0	0.77	0.82	5.4	10	9.8	0.81	0.82	5.3
DCA18	21	13	10.4	0.79	0.80	4.9	9	8.96	0.78	0.77	4.1	7	6.98	0.73	0.70	3.2	13	12.7	0.72	0.82	5.4
GAPU71A	5	4	4.00	0.64	0.64	2.7	5	5.00	0.61	0.69	3.1	4	4.00	0.70	0.69	3.1	3	3.00	0.46	0.44	1.8
DCA14	17	15	11.7	0.80	0.80	5.0	10	9.87	0.78	0.78	4.4	7	7.00	0.69	0.71	3.3	6	5.69	0.67	0.68	3.0
PA(ATT)2	3	2	2.00	0.15	0.16	1.2	3	3.00	0.26	0.29	1.4	2	2.00	0.13	0.13	1.1	2	1.98	0.04	0.04	1.0
DCA08	14	13	10.3	0.82	0.83	5.8	10	9.91	0.83	0.81	4.9	4	4.00	0.71	0.72	3.5	7	6.95	0.69	0.70	3.3
DCA09	14	13	9.33	0.53	0.57	2.3	7	7.00	0.78	0.77	4.1	10	9.82	0.67	0.72	3.4	8	7.80	0.81	0.73	3.6
DCA01	32	28	20.23	0.93	0.92	12.0	20	19.6	0.87	0.91	10.0	19	18.6	0.84	0.85	6.4	17	16.5	0.9	0.88	8.0
DCA15	3	2	2.00	0.21	0.21	1.3	3	2.96	0.28	0.24	1.3	2	2.00	0.47	0.50	2.0	2	2.00	0.27	0.49	1.9
DCA03	10	10	7.26	0.68	0.67	3.0	6	5.96	0.65	0.64	2.7	8	7.91	0.71	0.78	4.4	7	6.95	0.71	0.76	4.0
EMO03	17	15	11.3	0.84	0.81	5.3	7	6.96	0.67	0.78	4.3	8	7.95	0.67	0.78	4.3	8	7.99	0.69	0.68	3.1
GAPU45	6	5	4.48	0.63	0.67	2.9	5	4.96	0.67	0.64	2.7	3	3.00	0.47	0.52	2.1	3	3.00	0.43	0.38	1.6
**Mean**	14	12	9.13	0.66	0.66	4.57	8	7.93	0.67	0.68	4.05	7.2	7.11	0.64	0.66	3.52	7.2	7.03	0.62	0.62	3.50
**cpDNA**	20	15	10.97	-	0.72	3.64	4	4.00	-	0.44	1.20	3	3.00	-	0.59	2.36	4	3.99	-	0.46	1.81

Table S5. Plastid SSR (cpSSR) profile and frequency of the 20 haplotypes detected in central Sahara based on 15 polymorphic plastid loci (see Table S3b; Besnard et al. 2011). Thirteen loci revealed two alleles, while the two remaining showed three (locus 19) and four alleles (locus 1). Loci 9, 19, and 33 have been already used in Besnard et al. (2007). Freq. = Frequency.

**Haplotype code**	**Freq.**	**Plastid DNA loci**														
		**1**	**2**	**9***	**15**	**19***	**25**	**28**	**33***	**38**	**39**	**41**	**45**	**48**	**51-I**	**57-II**
E1-*l*1.1	0.405	12	9	21	15	33	13	12	11	11	13	11	10	22	18	15
E1-*l*1.2	0.128	12	9	21	15	33	13	12	12	11	13	11	10	22	18	15
E1-*l*1.3	0.198	13	9	21	15	33	13	12	11	11	13	11	10	22	18	15
E1-*l*1.4	0.041	12	9	21	15	33	13	12	11	11	13	11	10	22	18	14
E1-*l*1.5	0.005	12	9	21	15	33	13	12	11	11	13	11	10	21	18	15
E1-*l*1.6	0.003	12	9	21	15	33	14	12	11	11	13	10	10	22	18	15
E1-*l*1.7	0.022	12	9	21	16	33	13	12	11	11	13	10	10	22	18	15
E1-*l*1.8	0.003	12	9	21	16	33	13	12	12	11	13	10	11	22	18	15
E1-*l*1.9	0.011	11	9	21	15	33	13	12	11	11	13	11	10	22	18	15
E1-*l*1.10	0.016	11	9	21	15	33	13	12	11	10	13	11	10	22	18	15
E1-*l*1.11	0.011	11	9	21	15	33	13	12	11	11	13	11	10	22	17	15
E1-*l*1.12	0.005	11	9	20	15	33	13	12	11	11	13	11	10	22	17	15
E1-*l*1.13	0.005	11	9	20	15	33	13	12	11	11	13	11	10	22	18	15
E1-*l*1.14	0.016	11	9	21	15	32	13	12	11	11	13	11	10	22	18	15
E1-*l*1.15	0.003	11	9	21	15	33	13	12	11	11	13	11	10	22	18	16
E1-*l*1.16	0.003	10	9	21	15	33	13	12	11	11	13	11	10	22	18	16
E1-*l*1.17	0.087	11	9	21	15	34	13	12	11	11	13	11	10	22	18	15
E1-*l*1.18	0.033	11	9	21	15	34	13	12	11	11	12	11	10	22	18	15
E1-*l*1.19	0.003	11	10	21	15	34	13	12	11	11	13	11	10	22	18	15
E1-*l*1.20	0.003	11	9	21	15	34	13	13	11	11	13	11	10	22	18	15

* Plastid DNA loci already characterized in Besnard et al. (2007), but with different primers; locus 9 = *psb*K-*trn*S-poltyT/A; locus 19 = *ccmp5*; and locus 33 = *trn*T-L-polyT.

Table S6. Pairwise F_{ST} values between the 15 Ahaggar subpopulations based on plastid haplotypes (cpDNA) and 12 nuclear SSRs (nSSR). Abbreviations: T-Hamor = Tin-Hamor; Akerak. = Akerakar; Ahouna. = Ahounamat; Illennan. = Illenanene.

	Einzebib	Intounine	Tahassa	Hadriane	T-Hamor	Akerak.1	Akerak.2	Akerak.3	Ahouna.	Ilennane.	Isekram	Tonget	Adjelella	Taessa
**[cpDNA]**														
Intounine	0.0956													
Tahassa	0.0331	0.1539												
Hadriane	0.0804	0.1336	0.1561*											
Tin Hamor	0.3726*	0.2430	0.4759*	0.4399*										
Akerakar 1	0.0680	0.2973	0.1189	0.2800*	0.4897*									
Akerakar 2	0.1530*	0.4488	0.1947	0.3764*	0.6140*	0.0098								
Akerakar 3	0.0815	0.3001	0.0965	0.2696*	0.5775*	0.0108	0.0563							
Ahounamat	0.0246	0.1587	0.0534	0.2150	0.4802*	0.0168	0.0468	0.0196						
Ilennanene	0.1101	0.0304	0.1786	0.1074	0.3303	0.3273*	0.4706*	0.3279	0.2285					
Isekram	0.0976	0.3716	0.1210	0.2893	0.6288*	0.0284	0.0561	0.0064	0.0028	0.3975				
Tonget	0.1920	0.5494*	0.2344	0.3960*	0.7300*	0.0794	0.0179	0.1157	0.1604	0.5565	0.0135			
Adjelalla	0.0144	0.0067	0.0679	0.0774	0.2492	0.1407	0.2684	0.1660	0.0534	0.0355	0.2048	0.3435		
Taessa	0.0322	0.2138	0.0499	0.2097	0.5410*	0.0050	0.0606	0.0180	0.0244	0.1546	0.0043	0.1608	0.0956	
Tizouadj	0.2389	0.5952	0.2699	0.4433*	0.7624*	0.1121	0.0347	0.1583	0.2075	0.6047*	0.0278	0.0462	0.3852	0.2222
**[nSSR]**														
Intounine	0.0423													
Tahassa	0.0138	0.0305												
Hadriane	0.0022	0.0244	0.0094											
Tin Hamor	0.0193	0.0070	0.0136	0.0015										
Akerakar 1	0.0136	0.0386	0.0117	0.0155	0.0117									
Akerakar 2	0.0156	0.0390	0.0010	0.0220	0.0158	-0.0036								
Akerakar 3	0.0203	0.0491	-0.0053	0.0142	0.0114	-0.0004	0.0058							
Ahounamat	-0.0041	0.0243	-0.0045	-0.0005	0.0056	-0.0036	0.0072	0.0036						
Ilennanene	0.0338	0.0686	0.0385	0.0382	0.0511	0.0339	0.0589	0.0364	0.0544					
Isekram	0.0354*	0.0504	0.0165	0.0327*	0.0217	0.0307	0.0154	-0.0042	0.0071	0.0585				
Tonget	0.0191	0.0544	0.0110	0.0201	0.0196	0.0200	0.0239	0.0177	0.0100	0.0274	0.0326			
Adjelalla	0.0198	0.0439	0.0057	0.0191	0.0192	0.0140	0.0095	0.0156	0.0069	0.0499	0.0138	0.0159		
Taessa	0.0182	0.0534	0.0182	0.0151	0.0091	0.0094	0.0161	-0.0043	-0.0112	0.0548	0.0238	0.0349	0.0317	
Tizouadj	0.0378	0.0222	0.0178	0.0274	0.0243	0.0208	0.0275	0.0076	0.0261	0.0018	0.0226	0.0213	0.0240	0.0146

* significantly different from 0 ($p < 0.01$), based on 105’000 permutations.

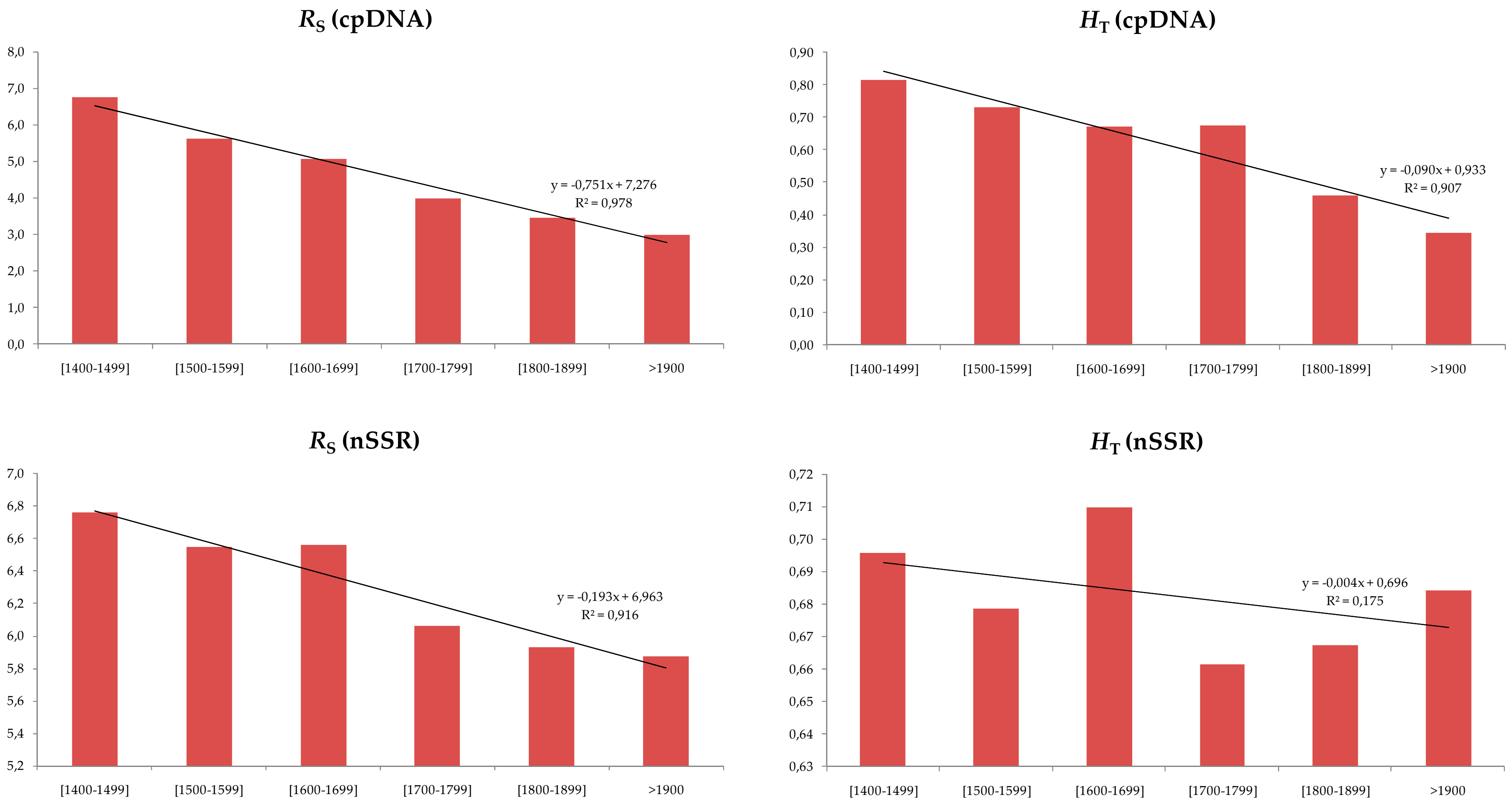

Figure S1. Mean allelic richness (R_S) and gene diversity (H_T) according to elevation within the Ahaggar massif assessed on chlorotypes (cpDNA) and nuclear microsatellites (nSSR). Six classes of altitudinal range were defined: (*i*) 1400 to 1499 m (63 individuals), (*ii*) 1500 to 1599 m (45 ind.), (*iii*) 1600 to 1699 m (41 ind.), (*iv*) 1700 to 1799 m (17 ind), (*v*) 1800 to 1899 m (55 ind.), and (*vi*) > 1900 m (11 ind.). A trend line (with a coefficient of determination) is given on each graph. For nSSRs, R_S and H_T were estimated on eight loci with no missing data: DCA01, DCA03, DCA05, DCA08, DCA09, DCA15, DCA18, and GAPU71A.

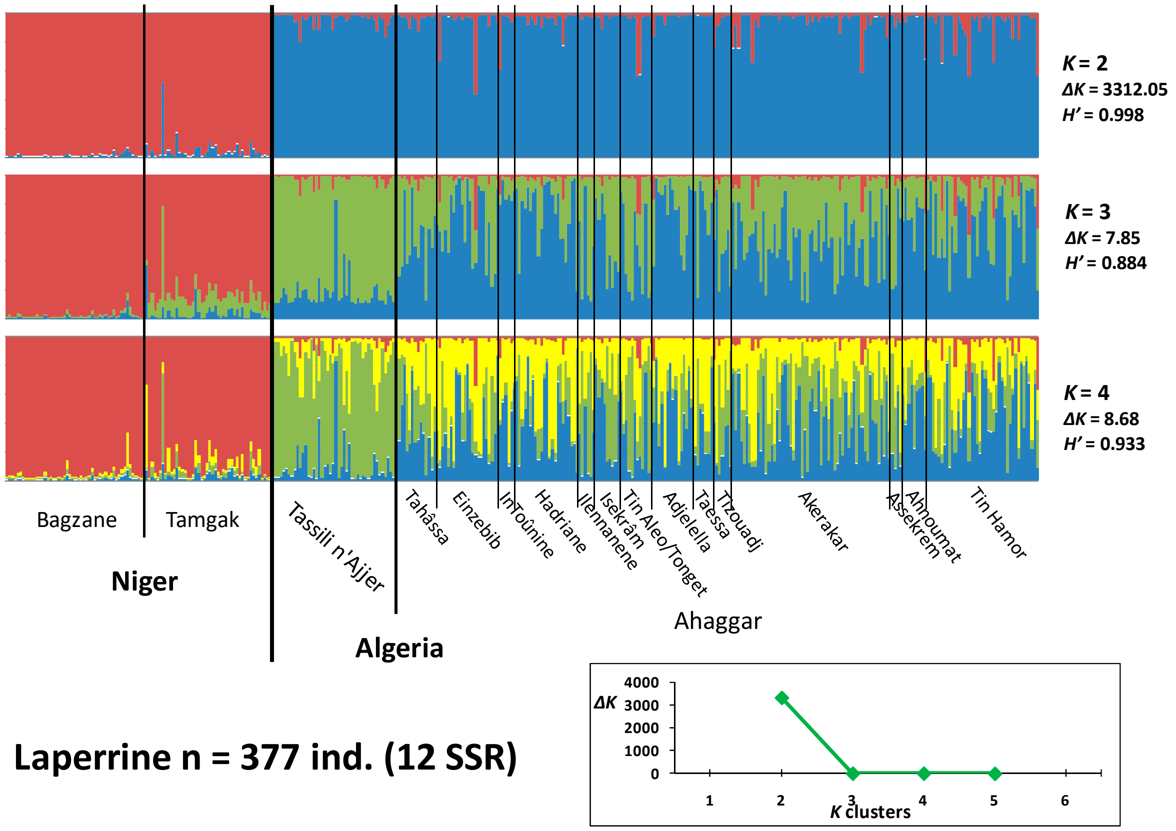

Figure S2. Inference of population structure using model-based Bayesian clustering implemented in STRUCTURE (Pritchard et al. 2000). This analysis was performed on the whole dataset (377 diploid genotypes; Table S3) based on 12 nuclear microsatellites (see Fig. S3 for the analysis excluding all individuals with missing data). The membership coefficient of assignment (*p*) of each individual to different gene pools is shown for $K = 2$, $K = 3$, and $K = 4$ clusters. H' represents the similarity coefficient between ten runs for each K value, and ΔK is the ad-hoc measure of Evanno et al. (2005). The graph at the bottom right gives ΔK plotted against K. The most likely genetic structure model is $K = 2$, according to ΔK and H'. At this K value, most individuals are correctly assigned to Niger or Algeria clusters. At $K = 3$, the Algerian cluster is split into two clusters suggesting a low differentiation between Tassili n'Ajjer and Ahaggar. At $K = 4$, clustering within the Ahaggar is unclear.

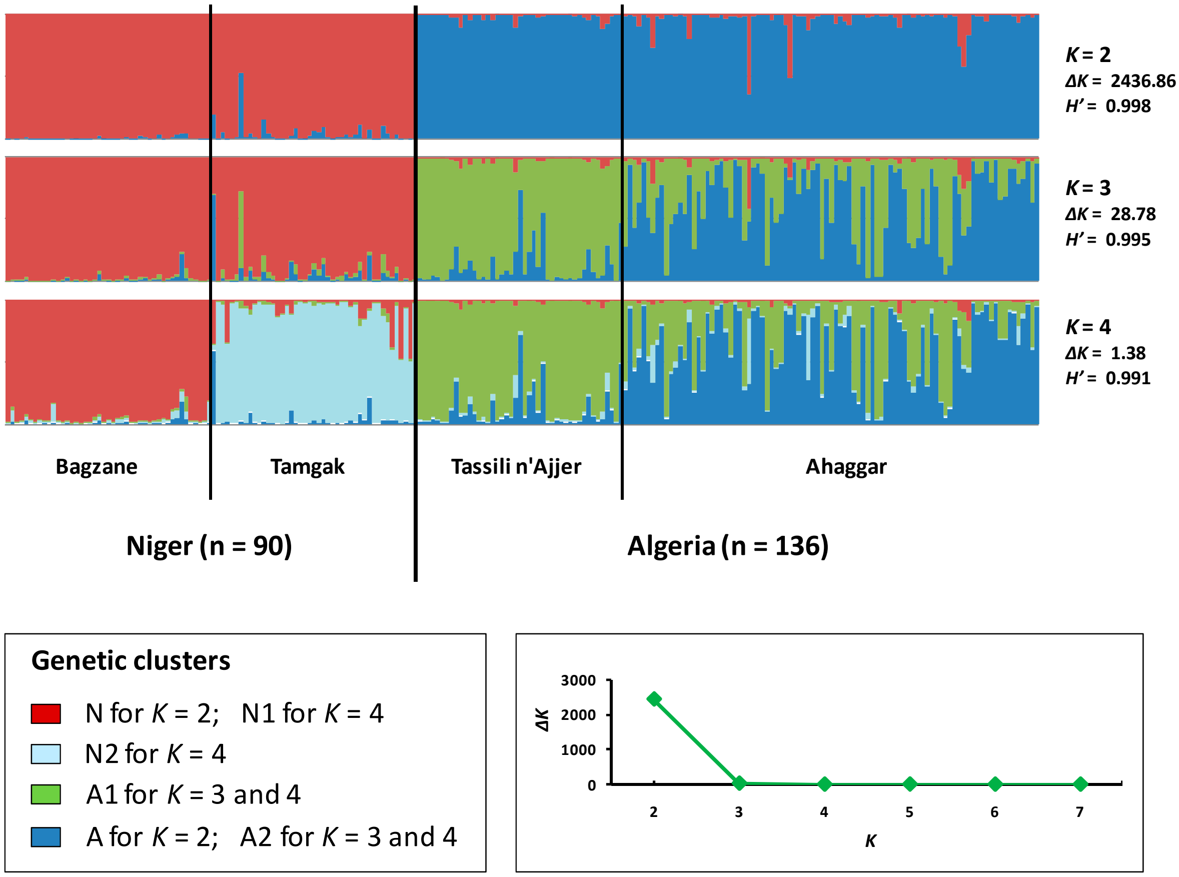

Figure S3. Inference of population structure on 226 individuals with no missing data using model-based Bayesian clustering implemented in STRUCTURE (Pritchard et al. 2000). The membership coefficient of assignment (p) of each individual to different gene pools is shown for $K = 2$, $K = 3$, and $K = 4$ clusters. H' represents the similarity coefficient between ten runs for each K value, and ΔK is the ad-hoc measure of Evanno et al. (2005). The graph at the bottom right gives ΔK plotted against K. The four main massifs are indicated as pre-defined regions. The most likely genetic structure model is $K = 2$, according to ΔK and H'. At this K value, most individuals are correctly assigned to Niger (N) or Algeria (A) clusters. At $K = 3$, the Algerian cluster is split into two clusters (A1 and A2) suggesting a low differentiation between Tassili n'Ajjer and Ahaggar. Finally, at $K = 4$, two gene pools are distinguished in Niger (N1 for Bagzane, and N2 for Tamgak).

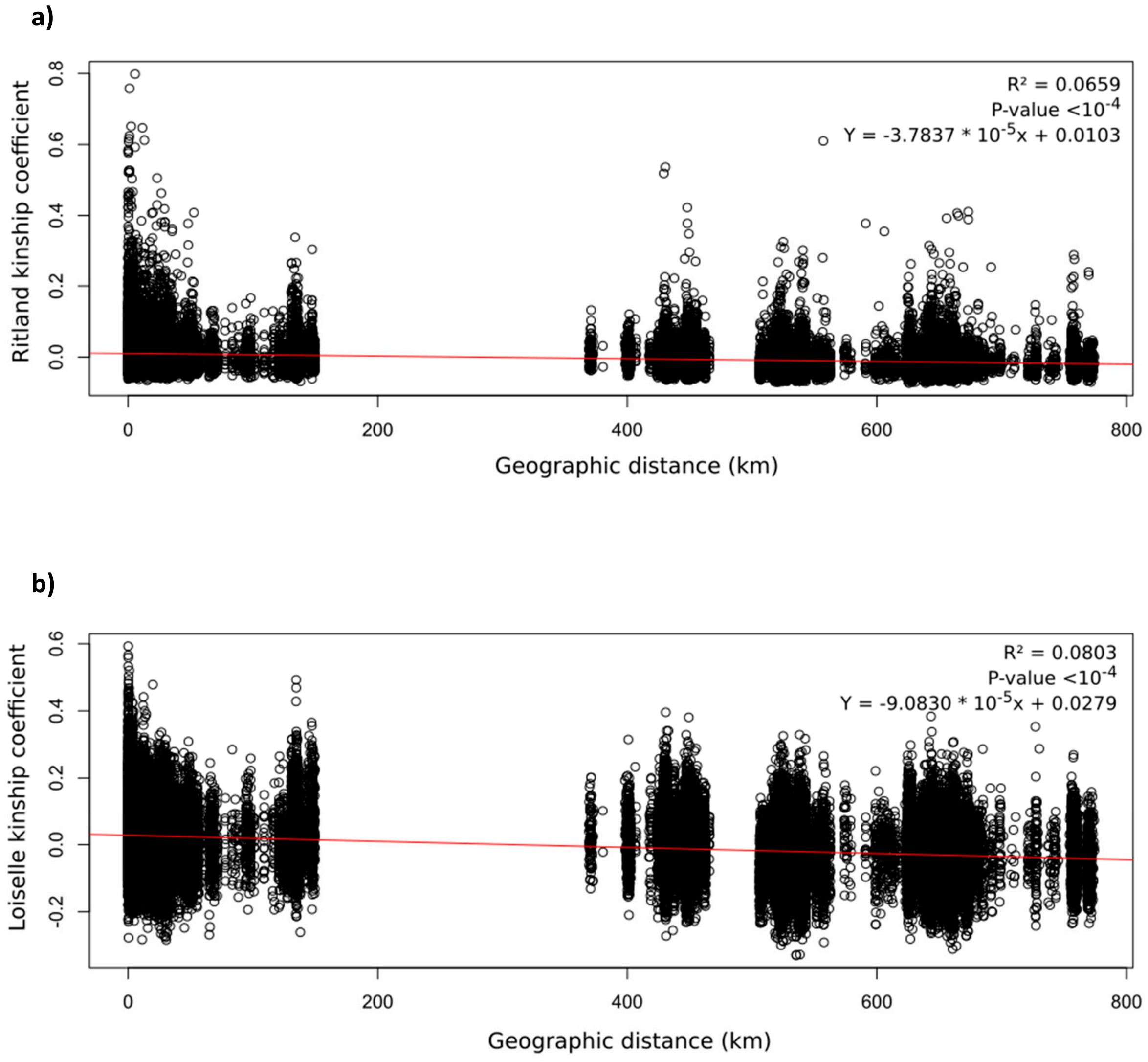

Figure S4. Pairwise kinship relationships according to geographic distance in Laperrine's olives of Central Sahara (North Niger and Southeast Algeria). The R-square and *p*-value statistics are given in the upper right. Each point represents a kinship estimate on a pair of individuals: **a)** for the Ritland coefficient (Ritland 1996), and **b)** for the Loiselle coefficient (Loiselle et al. 1995).

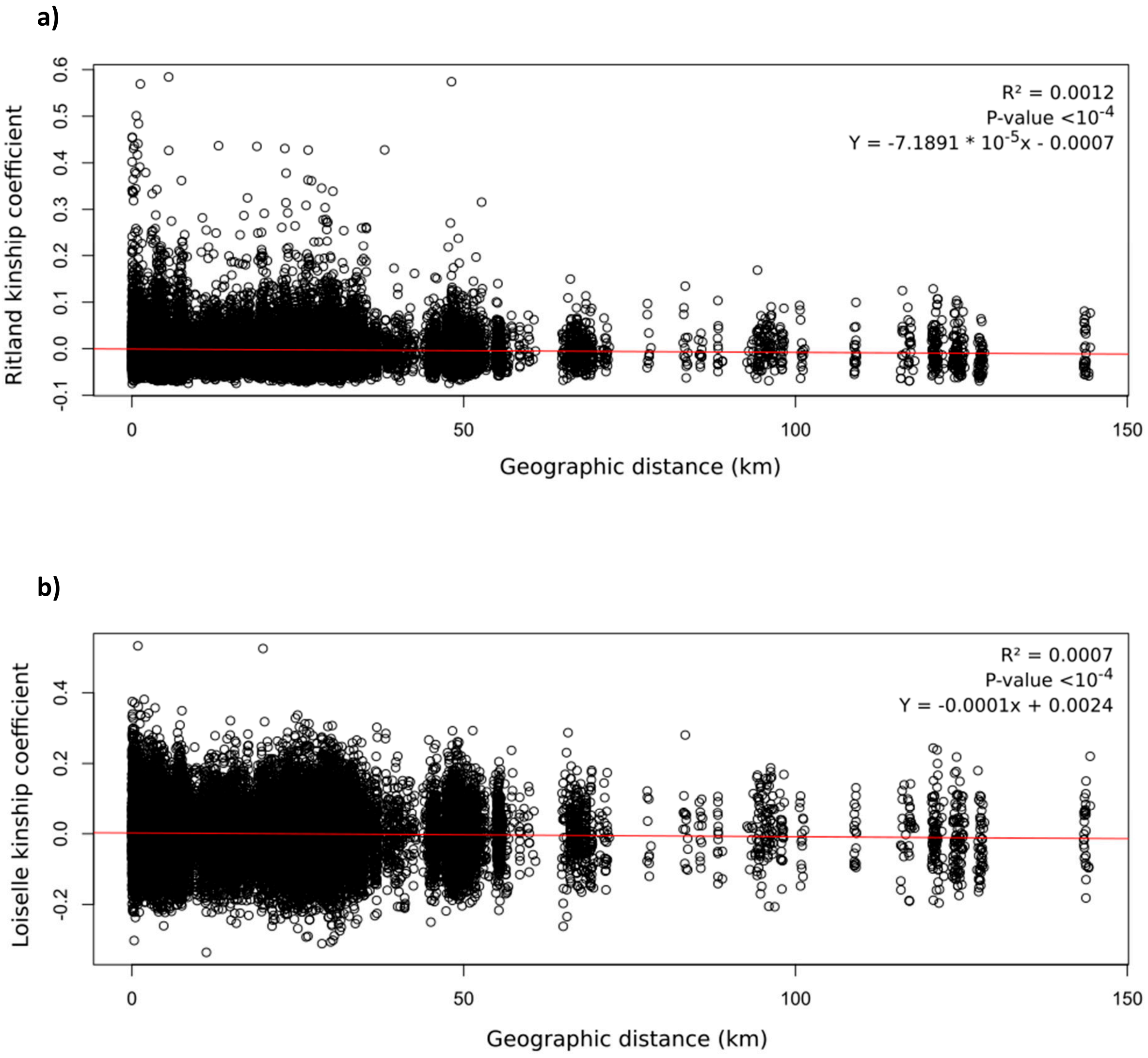

Figure S5. Pairwise kinship relationships according to geographic distance in Laperrine's olives of the Ahaggar massif (Southeast Algeria). The R-square and *p*-value statistics are given in the upper right. Each point represents a kinship estimate on a pair of individuals: **a)** for the Ritland coefficient (Ritland 1996), and **b)** for the Loiselle coefficient (Loiselle et al. 1995).

a)

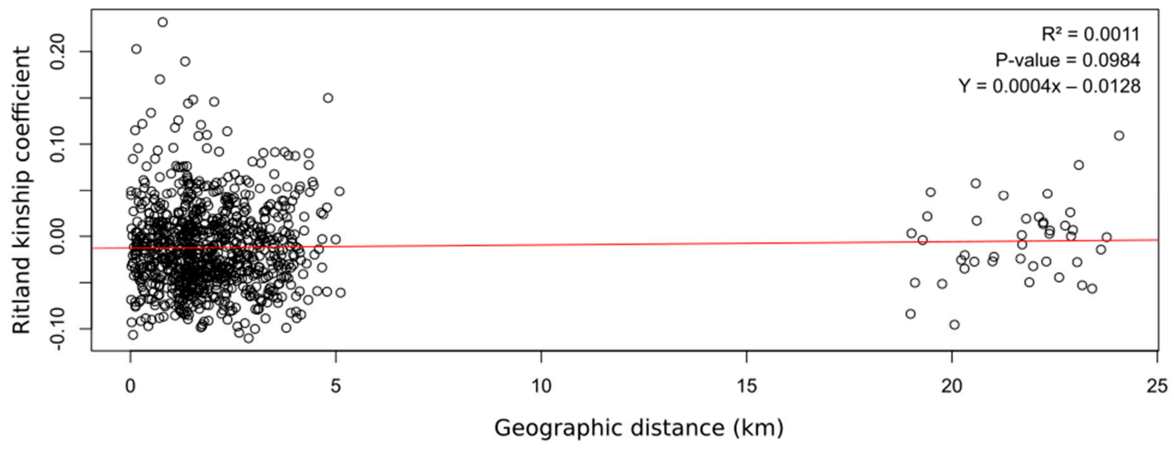

b)

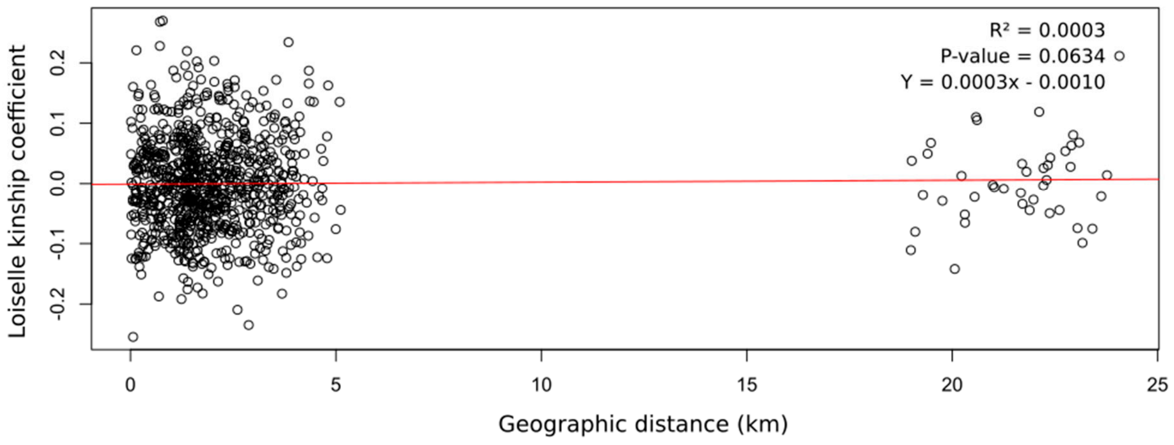

Figure S6. Pairwise kinship relationships according to geographic distance in Laperrine's olives of the Tassili n'Ajjer massif (Southeast Algeria). The R-square and *p*-value statistics are given in the upper right. Each point represents a kinship estimate on a pair of individuals: **a)** for the Ritland coefficient (Ritland 1996), and **b)** for the Loiselle coefficient (Loiselle et al. 1995).

a)

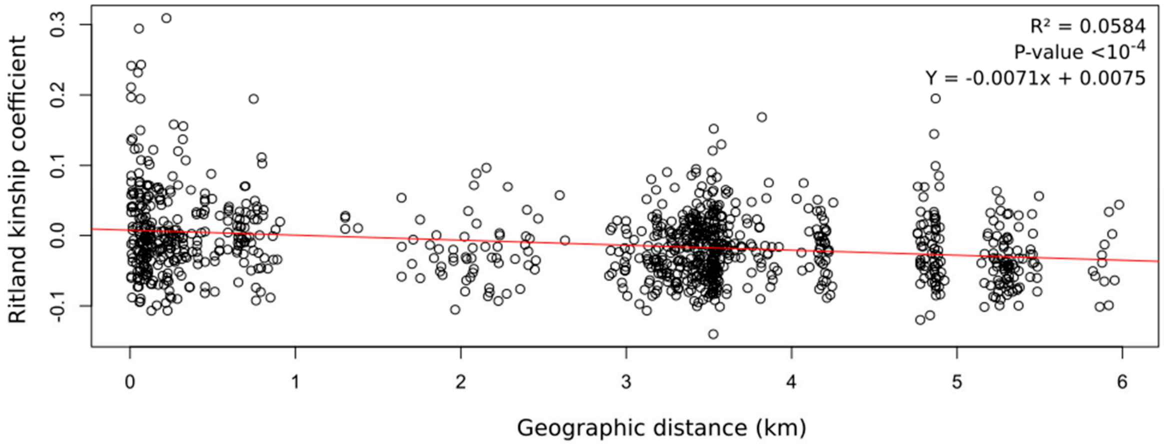

b)

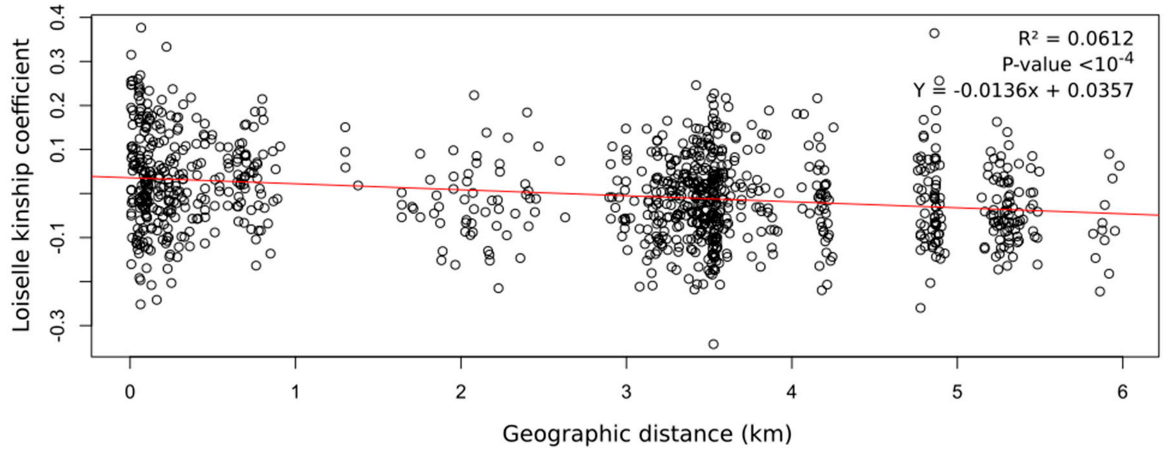

Figure S7. Pairwise kinship relationships according to geographic distance in Laperrine's olives of the Tamgak massif (North Niger). The R-square and *p*-value statistics are given in the upper right. Each point represents a kinship estimate on a pair of individuals: **a)** for the Ritland coefficient (Ritland 1996), and **b)** for the Loiselle coefficient (Loiselle et al. 1995).

a)

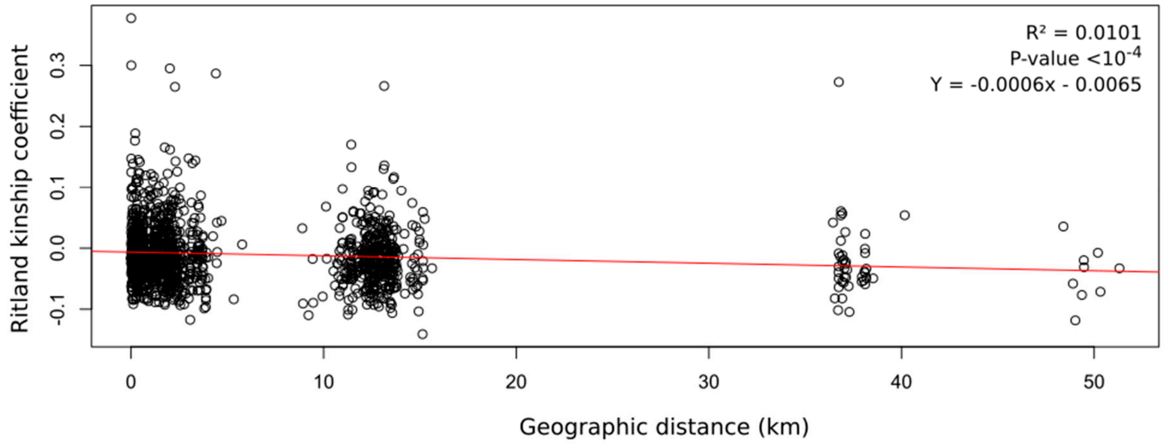

b)

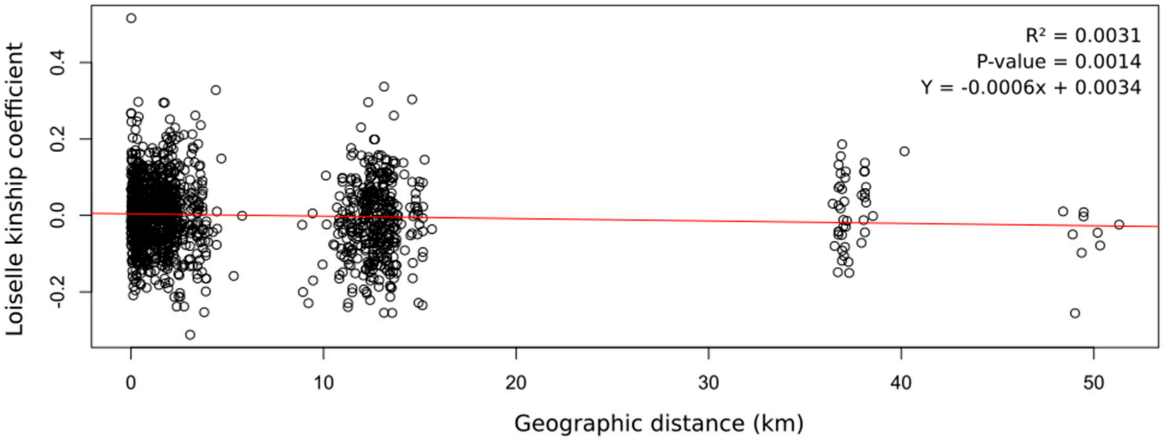

Figure S8. Pairwise kinship relationships according to geographic distance in Laperrine's olives of the Bagzane massif (North Niger). The R-square and *p*-value statistics are given in the upper right. Each point represents a kinship estimate on a pair of individuals: **a)** for the Ritland coefficient (Ritland 1996), and **b)** for the Loiselle coefficient (Loiselle et al. 1995).

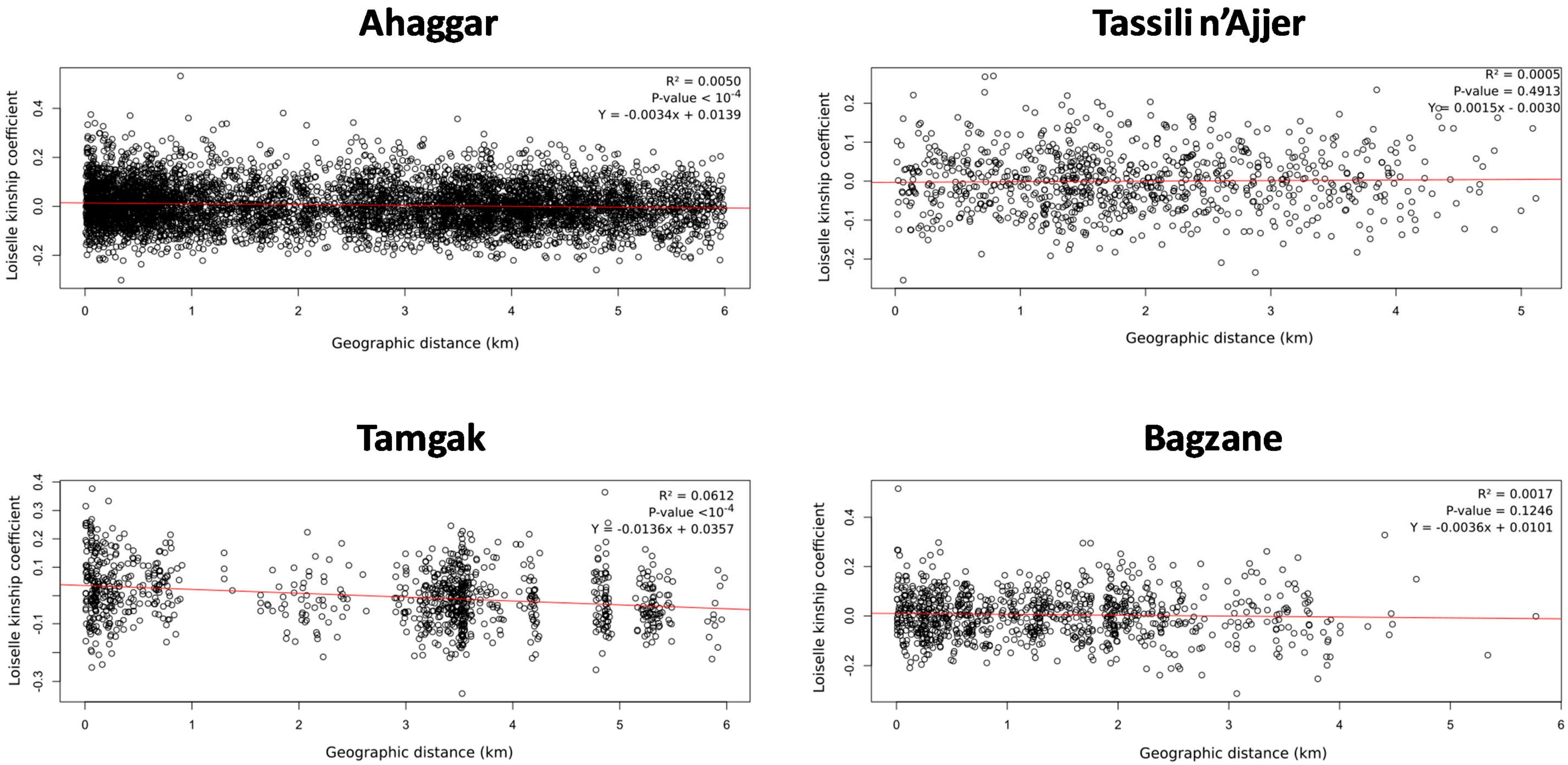

Figure S9. Comparison of pairwise kinship relationships (Loiselle et al. 1995) according to geographic distance in four Saharan massifs on a 6-km scale. The R-square and *p*-value statistics are given in the upper right. Each point represents a kinship estimate on a pair of individuals.

References

Besnard G, Baali-Cherif D (2009) Coexistence of diploids and triploids in a Saharan relict olive: evidence from nuclear microsatellite and flow cytometry analyses. *C. R. Biol.* 332:1115–1120.

Besnard G, Christin P-A, Baali-Cherif D, Bouguedoura N, Anthelme F (2007) Spatial genetic structure in the Laperrine's olive (*Olea europaea* subsp. *laperrinei*), a long-living tree from central-Saharan mountains. *Heredity* 99:649–657.

Besnard G, El Bakkali A (2014) Sequence analysis of single-copy genes in two wild olive subspecies (*Olea europaea* L.): nucleotide diversity and putative use for testing admixture. *Genome* 57:145–153.

Besnard G, Hernández P, Khadari B, Dorado G, Savolainen V (2011) Genomic profiling of plastid DNA variation in the Mediterranean olive tree. *BMC Plant Biol.* 11:80.

Evanno, G, Regnaut S, Goudet J. 2005. Detecting the number of clusters of individuals using the software structure: a simulation study. *Mol. Ecol.* 14:2611–2620.

Loiselle, BA, Sork VA, Nason J, Graham C (1995) Spatial genetic structure of a tropical understory shrub, *Psychotria officinalis* (Rubiaceae). *Am. J. Bot.* 82:1420–1425.

Pritchard JK, Stephens M, Donnelly P (2000) Inference of population structure using multilocus genotype data. *Genetics* 155:945–959.

Ritland K (1996) Estimators for pairwise relatedness and individual inbreeding coefficients. *Genet. Res.* 67:175–185.
